# Diagnostics and therapy of vestibular schwannomas – an interdisciplinary challenge

**DOI:** 10.3205/cto000142

**Published:** 2017-12-18

**Authors:** Steffen Rosahl, Christopher Bohr, Michael Lell, Klaus Hamm, Heinrich Iro

**Affiliations:** 1 Department of Neurosurgery, Helios Hospital of Erfurt, Germany; 2 Department of Otolaryngology, University Hospital of Erlangen, Germany; 3 Institute for Radiology and Nuclear Medicine, Hospital of Nuremberg, Germany; 4 Cyberknife Center of Central Germany, Erfurt, Germany

**Keywords:** vestibular schwannoma, acoustic neuroma, management, microsurgery, radiosurgery, quality of life

## Abstract

Vestibular schwannomas (VS) expand slowly in the internal auditory canal, in the cerebellopontine angle, inside the cochlear and the labyrinth. Larger tumors can displace and compress the brainstem. With an annual incidence of 1:100,000 vestibular schwannoma represent 6–7% of all intracranial tumors. In the cerebellopontine angle they are by far the most neoplasm with 90% of all lesions located in this region. Magnetic resonance imaging (MRI), audiometry, and vestibular diagnostics are the mainstays of the clinical workup for patients harboring tumors. The first part of this paper delivers an overview of tumor stages, the most common grading scales for facial nerve function and hearing as well as a short introduction to the examination of vestibular function.

Upholding or improving quality of life is the central concern in counseling and treating a patient with vestibular schwannoma. Preservation of neuronal function is essential and the management options – watchful waiting, microsurgery and stereotactic radiation – should be custom-tailored to the individual situation of the patient. Continuing interdisciplinary exchange is important to monitor treatment quality and to improve treatment results. Recently, several articles and reviews have been published on the topic of vestibular schwannoma. On the occasion of the 88^th^ annual meeting of the German Society of Oto-Rhino-Laryngology, Head and Neck surgery a special volume of the journal “HNO” will be printed. Hence this presentation has been designed to deviate from the traditional standard which commonly consists of a pure literature review. The current paper was conceptually woven around a series of interdisciplinary cases that outline examples for every stage of the disease that show characteristic results for management options to date. Systematic clinical decision pathways have been deduced from our experience and from results reported in the literature. These pathways are graphically outlined after the case presentations.

Important criteria for decision making are size and growth rate of the tumor, hearing of the patient and the probability of total tumor resection with preservation of hearing and facial nerve function, age and comorbidity of the patient, best possible control of vertigo and tinnitus and last but not least the patient’s preference and choice. In addition to this, the experience and the results of a given center with each treatment modality will figure in the decision making process. We will discuss findings that are reported in the literature regarding facial nerve function, hearing, vertigo, tinnitus, and headache and reflect on recent studies on their influence on the patient’s quality of life. Vertigo plays an essential role in this framework since it is an independent predictor of quality of life and a patient’s dependence on social welfare.

Pathognomonic bilateral vestibular schwannomas that occur in patients suffering from neurofibromatosis typ-2 (NF2) differ from spontaneous unilateral tumors in their biologic behavior. Treatment of neurofibromatosis type-2 patients requires a multidisciplinary team, especially because of the multitude of separate intracranial and spinal lesions.

Off-label chemotherapy with Bevacizumab can stabilize tumor size of vestibular schwannomas and even improve hearing over longer periods of time. Hearing rehabilitation in NF2 patients can be achieved with cochlear and auditory brainstem implants.

## 1 Introduction

Vestibular schwannomas are benign tumors growing typically in the internal auditory meatus and in the cerebellopontine angle. Still the term of acoustic neuroma is used, which is strictly speaking not correct, but has been coined wrongly in medical language. Today, this type of neuroectodermal tumor is called vestibular schwannoma because it develops from the Schwann cells of the vestibular root of the vestibulocochlear nerve [1], [2]. It is a slowly growing tumor and displaces other structures. In cases of bilateral occurrence, it is always defined as neurofibromatosis type 2 (NF-2) [3], [4], [5], [6]. These tumors have to be differentiated from vestibular schwannomas with regard to their biological and clinical characteristics. They show a highly variable growth behavior and are associated with other tumors, among others with gliomas and meningiomas [1], [7], [8]. Vestibular schwannomas represent about 6–7% of all intracranial tumors, however, with 90% they are the most frequent lesion appearing in the cerebellopontine angle [9]. The annual incidence amounts to 1:100,000. The tumor may appear generally in every age of life, but the main manifestation is between the 3^rd^ and 5^th^ decade [10]. Regarding the gender distribution, contradictory data are found in the literature. Brown et al. mention a majority of female patients and assume a hormonal process for the development of vestibular schwannomas [11]. Other authors do not find differences regarding the gender distribution [12], [13], [14].

In the focus of counseling and treatment of patients suffering from vestibular schwannomas is the permanent maintenance or improvement of the quality of life. Hereby, multiple factors play a crucial role, e.g. from organ-related symptoms such as hearing loss, functional disorder of the facial nerve, vertigo, vestibular disorders, tinnitus, via the fear of a growing tumor that had remained in situ or residual findings during decades of follow-up controls up to social factors such as invalidity as well as social isolation.

In principle, depending on the size, there is no imperative need to treat an asymptomatic, non-growing vestibular schwannoma. In contrast, for example the removal of a small tumor from the cerebellopontine angle is an excellent chance to preserve the hearing ability of an affected patient and thus his quality of life. The patient’s decision to undergo treatment or not depends on various factors and may be completely different for the same tumor entity and the same symptoms, even if the medical counselor transmits always the same information during the conversations. 

In the following, the diagnosis and classification of the disease will be described. This article does not include methodical-technical aspects of the treatment (microsurgical techniques, approaches, intraoperative monitoring, or dose determination in radiosurgery) in order to keep the manuscript coherent and clear. In this way, there was space for a systematic series of case examples that complete already published statistics by their clarity and present current possibilities and treatment outcomes descriptively. 

## 2 Imaging diagnostics

Magnetic resonance imaging (MRI) is the method of choice for detection, staging, and follow-up of vestibular schwannomas. The examination should be performed with high-field MR systems (1.5–3 Tesla field strength) with dedicated surface coils. A typical scan protocol consists of FLAIR (fluid attenuated inversion recovery), T1-weighted (T1-w) spin echo (SE) or turbo spin echo (TSE) before and after application of contrast media and thin-slice (=1 mm) T2-weighted 3D gradient echo (e.g. CISS = constructive interference in a steady state; FIESTA-C = fast imaging employing steady state; …), or 3D-TSE (e.g. SPACE = sampling perfection with application optimized contrasts using different flip angle evolution; VISTA = volume isotropic turbo spin echo acquisition; CUBE) sequences. A diffusion-weighted sequence can be performed optionally to exclude/confirm acute ischemia.

Mainstay is the thin-slice T2-w 3D sequence that shows high contrast between liquor on the one hand and nerves, vessels, and bone on the other hand and thus has a cisternographic effect. The acquired images can be re-formatted in arbitrary orientation because of the isotropic resolution. It is suitable for exact tumor volumetry in the follow-up as well as for preoperative assessment of the tumor in relation to the brainstem, the fundus, the vessels and nerves. Furthermore, the assessment of the inner ear is best achieved with this sequence type [15], [16], [17]. The high-resolution T1-w sequence before and after application of contrast agent facilitates the detection of very small tumors and the postoperative evaluation, especially the differentiation between scars and residual tumor tissue or recurrences. The FLAIR sequence that covers the entire neurocranium, is used as an overview sequence to exclude other pathologies. If appropriate, a T1-w sequence with contrast agent assessing the entire neurocranium can be added to detect lesions affecting the blood-brain barrier or meningiomas (e.g. in the context of NF2). In order to allow for an objectively well traceable follow-up of the tumor size, the examination protocol should be standardized and measurements should be saved in PACS.

## 3 Management of the disease

Regarding the management of the disease, numerous articles have been published, among others there is a recent review article in the German journal *HNO* that reflects the state-of-the-art based on the relevant international literature. 

Hence, we decided to focus in this article less on theory but mostly on practice and interdisciplinarity. In the past, many dogmas overshadowed the treatment of patients with vestibular schwannomas. In the times of microsurgery, for a long time the complete tumor resection was considered as most important to avoid tumor recurrences, sometimes even at the expense of functional preservation. The introduction of stereotactic irradiation and the associated possibility to achieve growth interruption of the tumor for a high percentage with at the same time a low rate of side effects, further increases the claims to microsurgical treatment. Today, the preservation of the neuronal function is most relevant. However, it must not be forgotten that this aim can often be achieved with complete tumor resection. For this reason, it is often not possible to develop ex ante the ideal treatment concept for an individual patient. Recurrences occur after surgery and also after irradiation; even bi-modal, combined treatments are not always successful.

**Figure 1:**
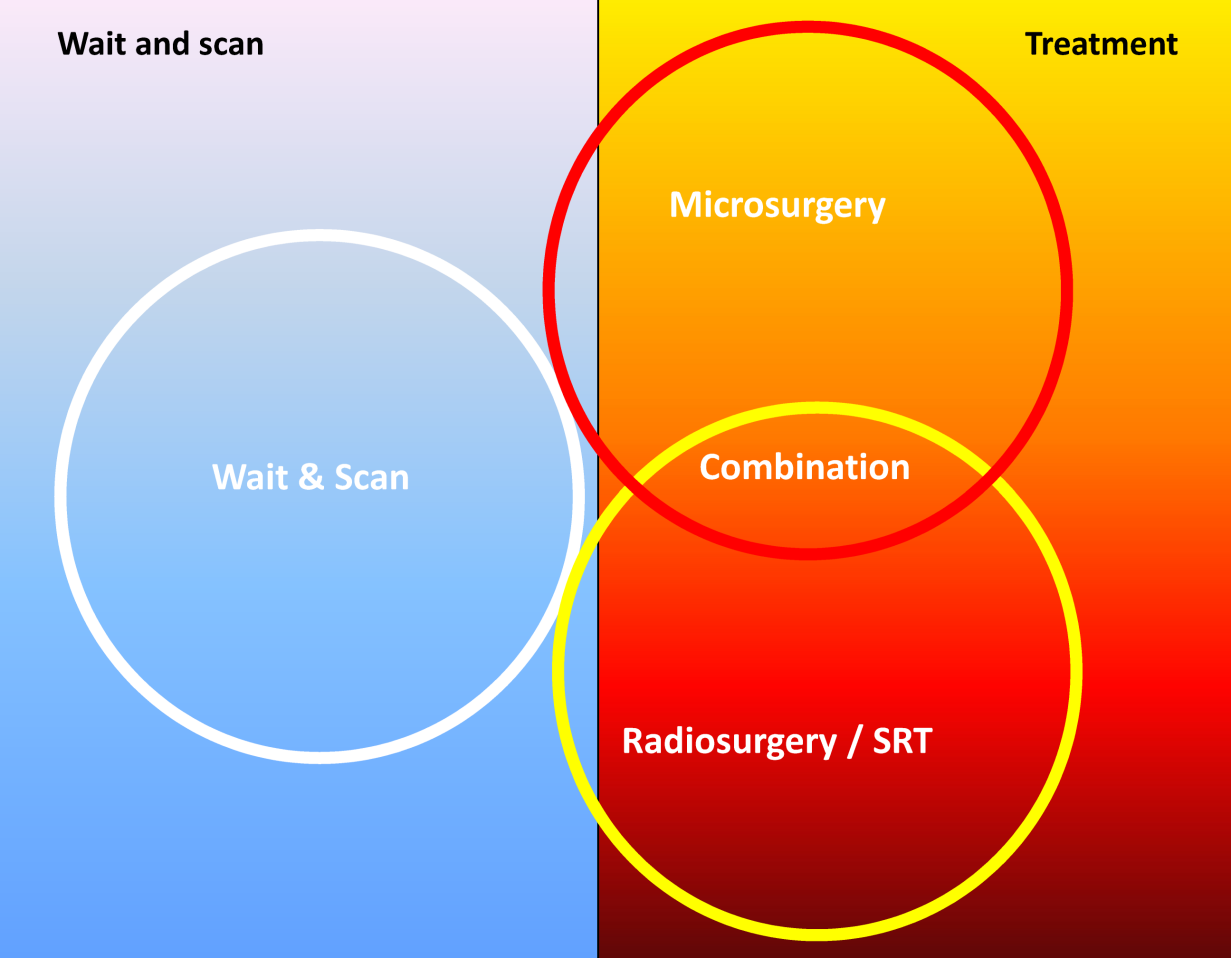
Options for the management of patients with vestibular schwannomas. Generally, for individual patients a combination of the 3 management pillars and at different times a switch from one modality to another is possible. So a primarily growing and then micro- or radiosurgically treated vestibular schwannoma may first be observed and in cases of recurrent growth, the other treatment procedure may be applied.

Even if a patient had a treatment outcome with a low risk of recurrences and good hearing, persisting tinnitus and vertigo might be remaining disorders that are difficult to tolerate. The management of the single patient will always require an individual concept that will have to be adjusted during the further course (Figure 1 (Fig. 1)).

This complexity makes it so difficult to create standardized treatment pathways for this disease. So it seems more important to establish a continuous interdisciplinary dialogue than to establish standards. Being open for treatment concepts that go beyond the control or removal of the tumor and that are oriented at the patient’s quality of life, as well as scientifically elaborating new diagnostic and therapeutic options will determine the future.

This present article was conceived around a case collection, in which the reader will find examples for every stage of the disease describing the current microsurgical and radio-therapeutic options of disease management. After the case series, suggestions are found for a classification regarding a reasonable and suitable management of the disease in the sense of decision cascades and clinical treatment pathways. Furthermore, factors are discussed that significantly influence the quality of life of patients suffering from vestibular schwannomas. There will also be an overview about the management of those tumors in patients with neurofibromatosis type 2. 

## 4 Tumor stages

**Figure 2:**
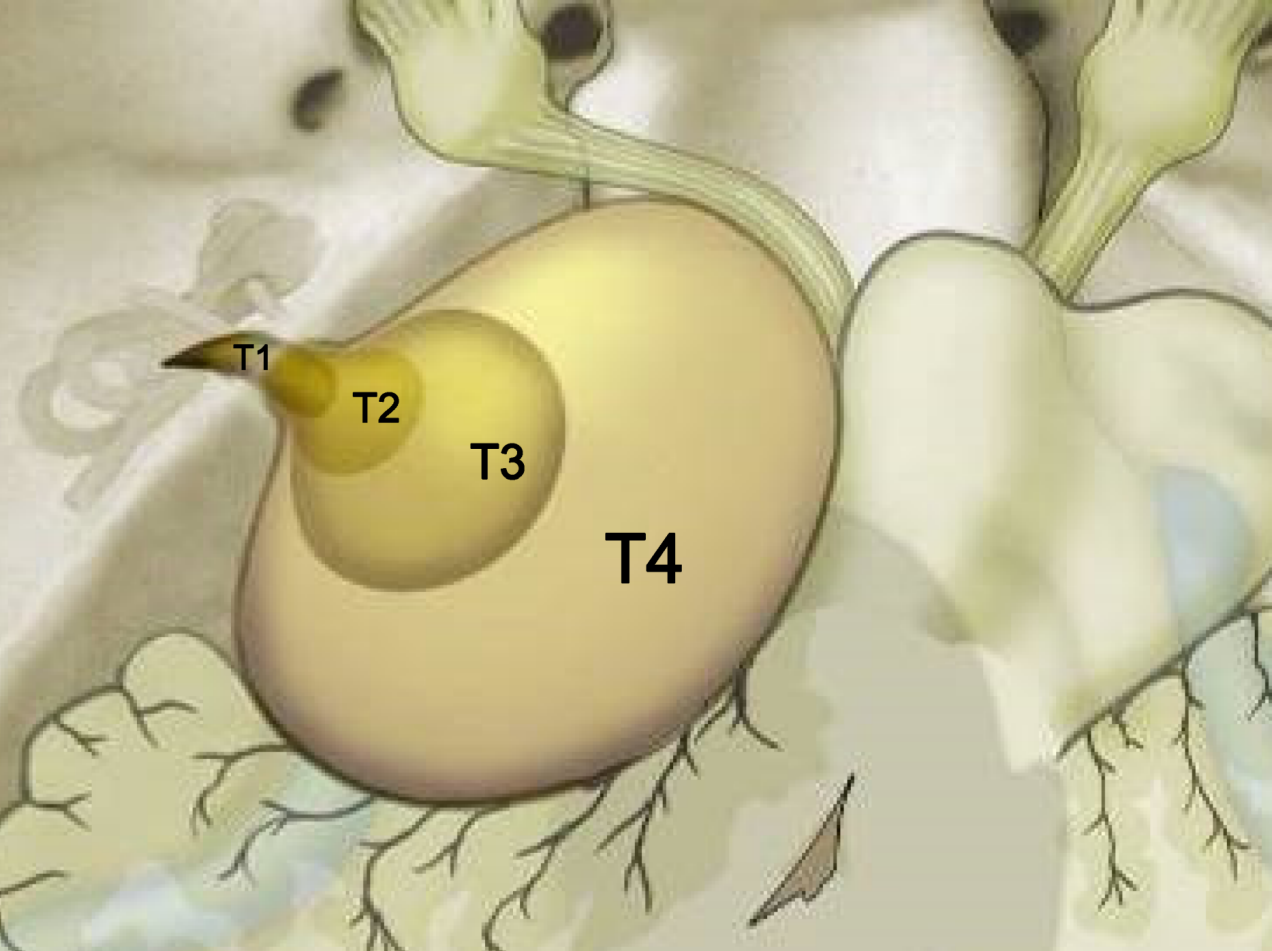
Tumor stages according to Samii [9, 20]. T1: intracanalicular; T2: intra-/extrameatal; T3: tumor reaches the brainstem; T4: tumor compresses the brainstem.

Tumor size and expansion play a crucial role in terms of the staging. Several authors have correlated these two criteria with different surgical approaches and treatment indications and outcomes [18]. As an example, the classification of Samii is cited here that – as the one of Koos [19] – differentiates mainly 4 tumor stages, however, especially patients in stage 4 are further differentiated regarding the presence of occlusion hydrocephalus [9], [20] (Figure 2 (Fig. 2)).

## 5 Classification of the hearing ability

As an example for a systematic classification of the hearing function, the guideline of the American Academy of Otolaryngology – Head and Neck Surgery (AAO-HNO) is mentioned [21]. It stages 4 “classes” of hearing ability; class A and B describe a still functional hearing that the patient may use in daily life. The classification is performed based on a pure tone audiogram, of which an average hearing loss is calculated over the frequencies 0.5, 1, 2, and 3 kHz (dB value in front of the decimal point) and based on a speech discrimination test indicating the percentage of words that are recognized at a volume of up to 40 dB above the hearing threshold or at maximally tolerable loudness (value behind the decimal point; normally hearing people recognize 100% of the words at 50 dB and more):

- Class A (≤30 dB, ≥70%)
- Class B (>30 dB ≤50dB, ≥50%)
- Class C (>50 dB, ≥50%)
- Class D (arbitrary, <50%)

According to this classification, functional hearing is only present in the classes A and B.

**Figure 3:**
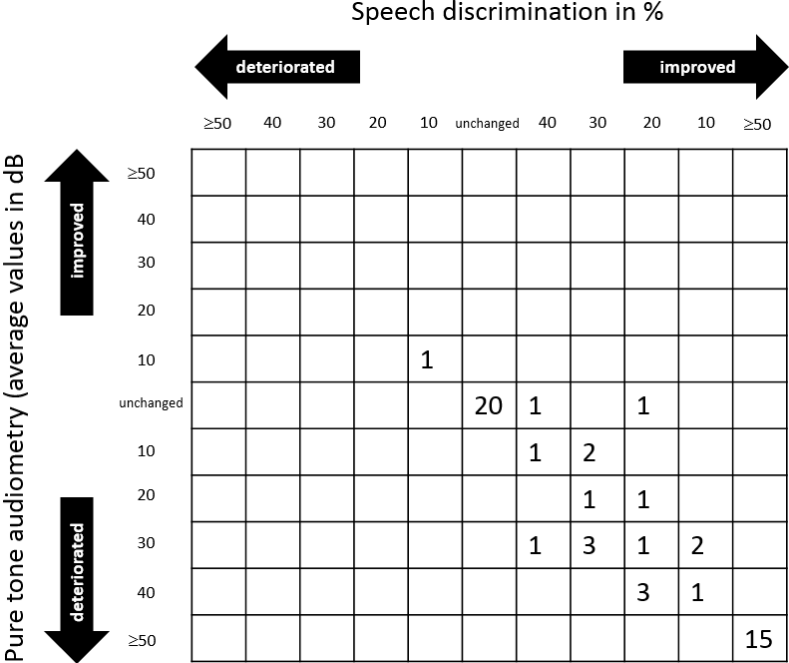
Cross-table with hypothetic data for displaying study results after interventions in cases of vestibular schwannomas. The columns represent the percentage of the changes of speech discrimination, the lines represent the average level changes in the speech range of the tone audiogram. An improvement of the hearing ability is improbable; hence the results are in the inferior left quadrant (modified according to [22]).

Regarding data collection in the speech discrimination test, standardization would be desirable, but currently it cannot be achieved. Ideally, a validated word list of at least 50 words in the patient’s mother language should be used, which is offered at a standardized level of 40 dB above the hearing threshold or maximally tolerable loudness [22]. In prospective studies or single centers, such a standardization is possible, in the practice still a certain heterogeneity of the data acquisition has to be tolerated. To describe the outcome regarding the hearing ability in a trial, pre- and postinterventionally established cross tables are an option. The columns display the speech discrimination and the lines the pure tone audiometry (Figure 3 (Fig. 3)). Such cross tables allow assessing the study results relatively clearly at a glance. 

## 6 Vestibular diagnostics

For the diagnosis as well as post-therapeutic follow-up of patients with vestibular schwannoma, vestibular diagnostics play a key role. In the context of pre-therapeutic examinations, the ENT-specialist is highly involved not only in the evaluation of the cochlear function and the function of the facial nerve, he is also responsible to find the exact diagnosis or the exclusion of a certain disease in cases of vertigo symptoms.

Even if nearly all patients report about the main symptom of unilateral hearing loss and tinnitus, about 10–15% of the patients suffer from initially unclear vertigo without hearing loss or tinnitus so that nearly all patients seek advice – sometimes by making some detours – of an otolaryngologist.

The low number of patients who initially complain about vertigo as single symptom and who are then diagnosed of vestibular schwannoma in the further course, may be explained by the slow growth of schwannomas and hereby sufficient time for central compensation.

If vertigo is observed, patients often describe unsystematic uncertain gait with possible increase in the dark or drift tendency to one side; frequently, they only mention it when they are specifically asked. An exact anamnesis of possible vertigo complaints requires sufficient time and attention as every vestibular test does, i.e. also for patients with vestibular schwannoma.

In particular after surgical therapy of vestibular schwannoma, patients report about acute vertigo attacks and postural instability. The intensity and severity of the postoperative vertigo is directly proportional to the preoperative vestibular function that has to be determined before surgery by means of extensive vestibular testing.

After clinical examination with Frenzel goggles, those examinations encompass also video-nystagmography at rest or after provocation by head shake, orientation and position test under video-nystagmography, caloric test, video head impulse test, examination of the vestibular evoked myogenic potentials as well as an examination in the rotating pendulum chair and an extensive functional testing, for example by means of sensory organization test.

Such a complete test battery requires time and staff and a good cooperation by the patient. If those preconditions are not fulfilled or available, the video head impulse test with testing all semicircular canals together with the VEMP testing of sacculus and utriculus already provides a good and time-efficient overview of the function of the peripheral vestibular organ performed by an experienced examiner and allows possible conclusions regarding the already damaged part of the vestibular nerve. 

In this way, the exact condition of the actual peripheral vestibular function can be measured and a possibly existing central affection in cases of large vestibular schwannomas as well as an already existing compensation can be revealed. Thus a good estimation of the functional postural situation is obtained.

Especially patients with bilateral vestibular problems can be identified who have to expect a severe and complicated postoperative course of the disease. In those cases, special preoperative information and counseling have to take place. If necessary, surgery of a vestibular schwannoma has to be postponed or it seems to be more appropriate that the patient undergoes radiotherapy. On the other hand, it is useful that patients with single-sided preoperative vertigo concerning the side of the schwannoma rather undergo surgical therapy because after transection of the vestibular nerve in the context of resection a better central compensation may probably be achieved. So extensive vestibular diagnostics also play a crucial role for making the best therapeutic decision. 

But also the possibility to inform patients about the expected postoperative complaints gains in importance with regard to current medico-legal aspects.

In general, the vestibular rehabilitation should be introduced very early in the postoperative phase and the patients are invited to undertake as many activities as possible that occupy the vestibular system. It is recommended to provide specific exercise instructions teaching the patients how to include balance exercises as often as possible in their daily life and to perform those exercises also with closed eyes to exclude the merely visual compensation. Also professional physiotherapy and exercise instructions as well as training by means of sensory neuro-feedback methods seem to be beneficial to achieve a rapid central compensation of postoperative vertigo.

In this way, the re-integration into daily life can be achieved rapidly and in general the patients are free of complaints after 6–9 months.

Beside the regular follow-up of the cochlear function, hearing rehabilitation, and imaging, also a short standardized checkup of the vestibular function should be performed in order to document a course of central compensation in the years after therapy. In cases of deterioration, possibly developing recurrence etc., vestibular diseases may be identified earlier.

In summary, preoperatively, the high value of sufficient vestibular diagnostics allows better information and counseling about different therapeutic options and expected side effects; and after therapy, the central objective to provide a quality of life as high as possibly can be achieved.

## 7 Function of the facial nerve

In the context of larger tumors, the function of the facial nerve is often impaired, which has an influence on the treatment approach. To assess the degree of neural paresis, the classification according to a proposal of William House and Derald Brackmann from 1985 could be internationally established (Table 1 (Tab. 1)).

Regarding further pre- and intraoperative diagnostic procedures (e.g. electrophysiological examination procedures such as acoustically evoked brainstem potentials) extensive literature on this topic has been published and will not be further discussed here. 

## 8 Surgical approaches

### 8.1 Transtemporal approach (middle fossa approach)

The transtemporal approach via the middle fossa is suitable for intrameatal and low-grade extrameatal tumors up to a size of 20 mm and patients with residual hearing [23]. The patient is lying in supine position, the head is slightly overstretched and turned to the contralateral side. Preauricular incision is performed starting from the level of the tragus nerve up to about 5 cm above the auricle in the temporal region. After preparation of the fascia of the temporal muscle and craniotromy, the temporal flap can be lifted and the internal auditory meatus is exposed in top view of the temporal bone and can be opened [24]. In neurosurgery, it is called the subtemporal approach because it refers to the temporal flap of the brain which should not be touched during surgery if possible.

### 8.2 Translabyrinthine approach

The translabyrinthine approach is suitable for patients with tumors that have already destroyed the hearing ability. Beside dissection of the internal auditory meatus, mastoidectomy as well as labyrinthectomy are performed. The advantages are the secure exposition of the facial nerve and the overview of the posterior fossa. The patient is lying in supine position and the head is turned to the contralateral side. After retroauricular, C-shaped incision and mastoidectomy exposing the sigmoid sinus, the dura of the posterior fossa as well as the facial nerve in its bony canal in the area of the second knee, and the labyrinthine bloc, first the lateral semicircular canal is drilled out, then the posterior and finally the superior semicircular canals follow. Afterwards, the vestibulum can be opened and the internal auditory meatus is exposed. The exposition and opening of the dura are performed until the intervention reaches the cerebellopontine angle [25], [26].

### 8.3 Retrosigmoid approach 

The retrosigmoid approach is especially suitable for tumors with larger extrameatal parts. It allows a secure and wide exposition of the posterior fossa and thus tumor resection with hearing preservation [27]. The facial nerve is exposed in the internal auditory meatus and at the brainstem, its further course at the ventral tumor side is only visible in the last step of the preparation [28], [29], [30]. In cases of smaller tumors, the patient is lying in supine position, the head is turned to the contralateral side and slightly inclined to the breast. In cases of larger tumors, the surgery site often has to be continuously rinsed and the irrigation fluid and the blood have to drain off, so that the patient may be positioned in a beach chair position; however, hereby the risk of air embolism is rather high [31]. The incision describes a bow and starts above the auricle and ends behind the mastoid tip. After exposition of large emissary veins during craniotomy, the incision of the dura is performed alongside the sigmoid and transverse sinus.

After opening the arachnoid mater in the area of the cerebellomedullaris cistern and draining off liquor, the cerebellopontine angle is exposed and the internal auditory meatus can be opened.

## 9 Illustrating cases

Instead of repeating findings that are published in the literature in a dry and manual-like manner, this contribution wants to convince mainly with a clear description of a case series and give ideas and hints for optimal information and treatment of affected patients.

According to the title of this article, those are exclusively cases in which at least the course of the disease justified treatment. Of course, especially in the context of older patients, most centers will first observe the spontaneous course by regularly performing clinical and imaging examinations. The annual – non-volumetric – growth rates of the tumors reported under strict observation varied between 0.3 and 4.8 mm [32], [33], [34]. Interestingly, the growth rate of a tumor seems to be a more reliable predictor for threatening hearing loss than the absolute tumor size at the time of diagnosis. In a meta-analysis of 982 patients, Sughrue et al. (2010) found out that patients with a tumor growth of less or equal 2.5 mm had a clearly higher preservation rate for their hearing ability (75% vs. 32%, p<0.0001), independent from the original size of the tumor [35].

If the indication for treatment is made in patients that were initially observed, it occurs in 75% of the cases within the first 5 years [32].

In order not to extend the volume of this article, spontaneous courses will not be included in the case series. Some spontaneous courses of the disease, however, will be described based on tumors that were treated later. Furthermore, a differentiation was made between primarily surgical and primarily radiotherapeutic cases in order to demonstrate as clearly as possible the possible effects of the treatment modalities. 

The examples of this case series are systematically classified according to the functional hearing abilities of the patients and previous treatment (surgery or irradiation). 

### 9.1 Microsurgery

#### Stage 1

**Functional hearing ability**

**Case #1, male, age: 52 years**

History:

- Vertigo independent from movement and tinnitus as permanent, high frequency whistling sound on the left side for several months
- Hearing unchanged
- Intracanalicular tumor confirmed by MRI, slightly grown after 6 months

Clinical findings:

- Paresis of the buccal branch of the facial nerve on the left side (House-Brackmann grade II), no other deficits
- Hearing: class A
- Caloric reflex test: side difference of 31% on the left side

**Figure 4:**
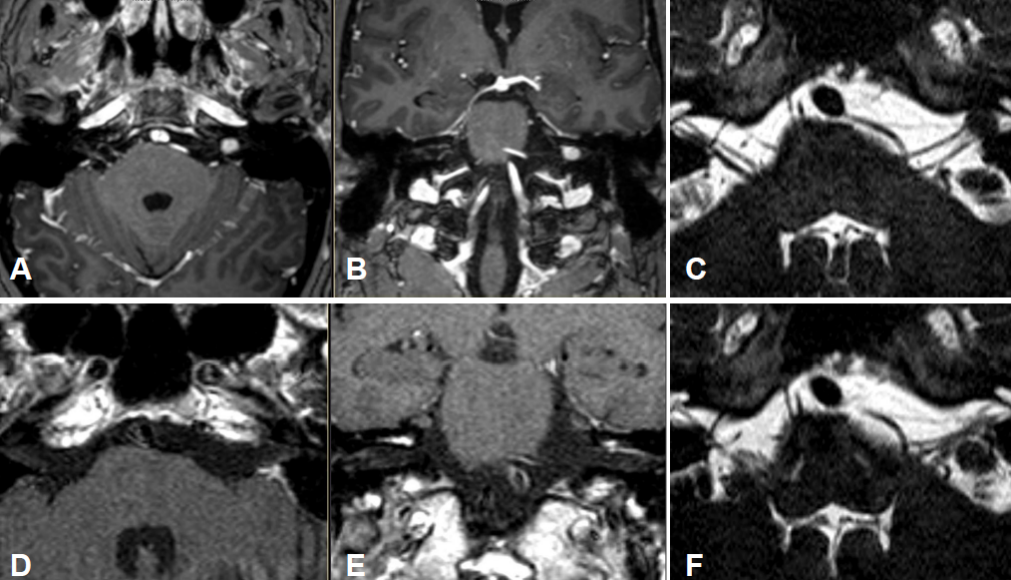
Upper row: preoperative MRI, T1-w + CM, A transversal; B coronal; C CISS transversal. Lower row: postoperative MRI, T1-w + CM; D transversal; E coronal; F transversal CISS.

MRI:

- Merely intracanalicular left-sided vestibular schwannoma (Figure 4 (Fig. 4))

Intervention:

- Complete tumor resection by microsurgery with preservation of the BAEP, the inferior vestibular nerve, and the facial nerve, performed via a retrosigmoid (lateral-suboccipital) approach.

Course:

- Postoperatively, increased vertigo for 3 weeks, especially in the context of lateral eye movements
- Facial nerve similar to preoperative findings
- Class A (according to AAO-HNO classification)
- Tinnitus slightly regressive, in particular at night
- 3 years after surgery, no recurrences

##### Stage 1

**Functional hearing ability**

**Case #2, male, age: 33 years**

History:

- First presentation with a history of right-sided progredient vestibular schwannoma known for 4 years

Clinical findings:

- Recurrent vertigo
- Vestibular test: well compensated, chronic-peripheral vestibular disorder of the right side
- Hearing: Class A, no tinnitus

**Figure 5:**
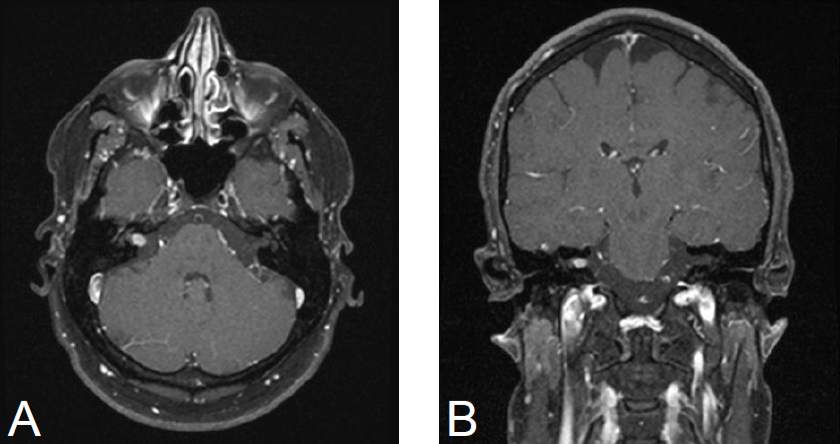
MRI, T1 FS + CM; A axila; B coronal. Intra- und slightly extrameatal vestibular on the right measuring 9x5x5 mm.

**Figure 6:**
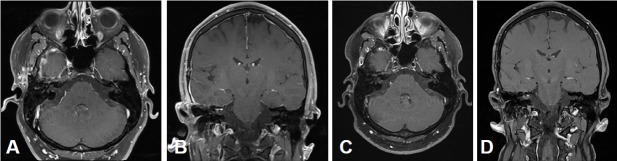
MRI, T1 FS + CM; A, C axial; B, D coronal; A, B 3 months after surgery; C, D 5 years after surgery. No hint for residual or recurrent tumor in the internal auditory meatus on the right side. Postoperative changes in the right temporal region.

MRI:

- Intra- and minimally extrameatal vestibular schwannoma located near the fundus, measuring 9x5x5 mm (Figure 5 (Fig. 5) and Figure 6 (Fig. 6))

Intervention:

- Transtemporal resection with monitoring of the facial nerve and intraoperative BERA on September 16, 2010

Course**:**

- No postoperative vertigo
- Facial nerve: House-Brackmann grade II; normalization within 8 weeks
- Class A; unchanged compared to surgery
- No tinnitus
- 5 years after surgery, no recurrences (Figure 6 (Fig. 6))

**No functional hearing ability**

**Case #3, male, age: 47 years**

History:

- In May 2013, the patient woke up in the morning with an acute vestibulo-cochlear disorder on the right side. First, severe rotational vertigo with subjective deafness and significant tinnitus on the right side. During therapy with cortisone, the tinnitus improved and the vertigo complaints were regressive. The attempt to perform external round window covering on the right side did not lead to improvement of the deafness.

**Figure 7:**
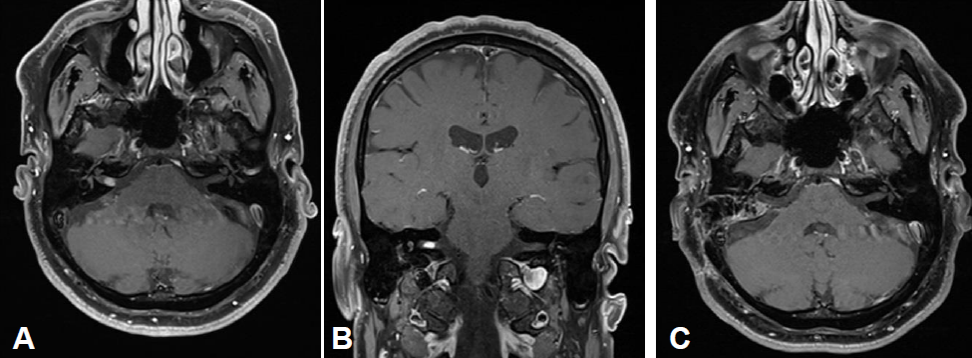
Preoperative MRI, T1 + CM; A axial; B coronal; 6x4x3 mm measuring intrameatal vestibular schwannoma on the right side. MRT, T1 + CM axial; C 5 months after surgery no hint for residual tumor or recurrent after translabyrinthine resection.

Clinical findings:

- Massive cochlea-vestibular disorder with labyrinth failure and right-sided deafness. Inconspicuous status of the vestibular nerve (Figure 7 (Fig. 7))

Intervention:

- Translabyrinthine resection of the vestibular schwannoma on the right side with monitoring of the facial nerve and insertion of a Contour Advanced dummy into the cochlea on January 28, 2014.

Course:

- Postoperative status of facial nerve: House grade I, slight vertigo with discrete spontaneous nystagmus to the left
- In the Dyna-CT scan dated January 29, 2014, the dummy was correctly located in the cochlea
- In the further course, rapid compensation of the vertigo with constant status of the facial nerve
- MRI on May 23, 2014 without suspicion for residual tumor or recurrence. Performance of a positive promontory test

##### Stage 2

**No functional hearing ability**

**Case #4, female, age: 36 years**

History:

- Hearing impairment for several months
- Phone calls are possible using the right ear
- Intermittent vertigo
- Significant tumor growth over 6 months

Clinical findings:

- Romberg’s and Unterberger tests are inconspicuous
- Audiometric high frequency hearing loss in both ears in the range of 2 kHz, on the right side to –60 dB, on the left side to –35 dB
- Speech discrimination of 70% on the right side at 60 dB

**Figure 8:**
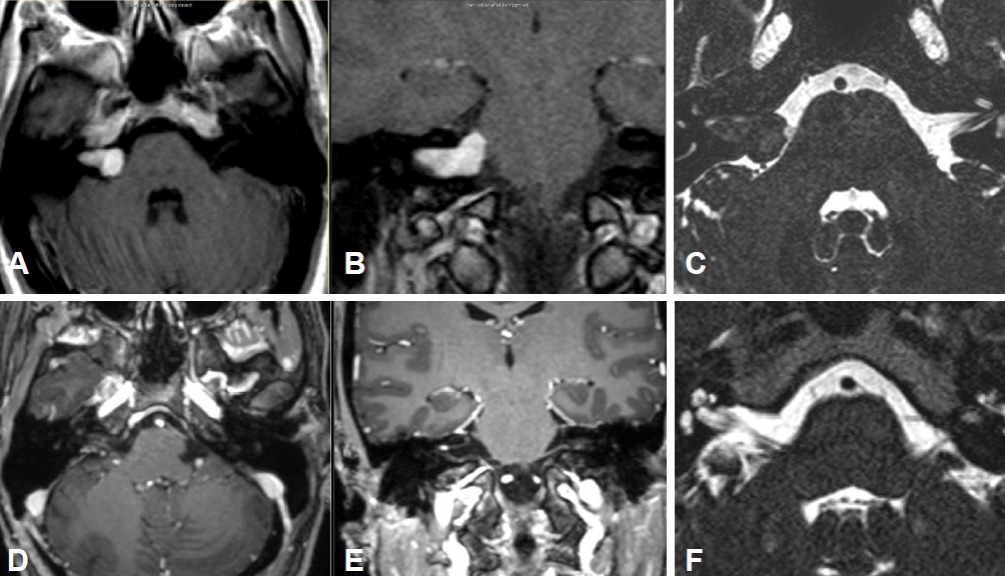
Upper row: preoperative MRI, T1-w + CM; A transversal, B coronal, C CISS transversal. Lower row: postoperative MRI, T1-w + contrast agent, D transversal; E coronal; F CISS transversal.

MRI:

- Inhomogeneous, intra-/extrameatal tumor on the right side that has not yet reached the fundus (stage T2). CISS images clearly demonstrate a thin layer of CSF between the tumor and the brainstem (Figure 8 (Fig. 8))

Intervention:

- Functional hearing preservation with complete microsurgical resection via a retrosigmoid (lateral-suboccipital) access. Functional preservation of the facial nerve (House-Brackmann grade 1)

Course:

- Increased vertigo for 3 weeks after surgery, especially in the context of lateral eye movements
- Slightly regressive tinnitus, in particular no impairment at night
- As preoperatively, the facial nerve is inconspicuous
- Class A (according to AAO-HNO)
- 3 years after surgery, no recurrence

**No functional hearing ability**

**Case #5, female, age: 55 years**

History:

- First presentation with known functional deafness and recurrent rotational vertigo since several months.
- MRI of the cerebellopontine angle performed in another institution confirmed the suspected vestibular schwannoma of the right side.

**Figure 9:**
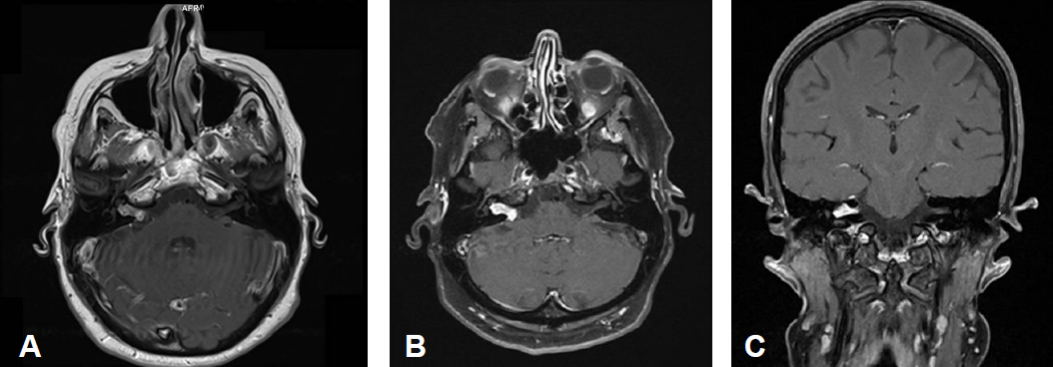
MRI, ; A axial, initial findings; T1 FS + CM, control after 4 months without therapy (B axial, C coronal).

**Figure 10:**
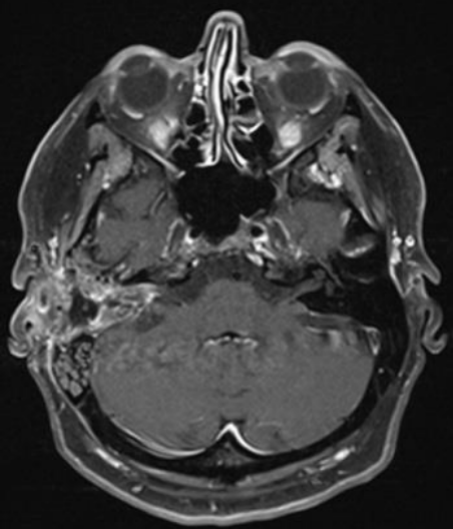
MRI 3 months after surgery, axial, T1 FS + CM. No recurrence after translabyrinthine resection 3 months after surgery.

Clinical findings:

- Occasional rotational vertigo and high-grade hearing loss in the right ear with inconspicuous status of the facial nerve
- Vestibular diagnostics: chronic peripheral vestibular disorder of the right side without complete central compensation
- Hearing: Class D (Figure 9 (Fig. 9) and Figure 10 (Fig. 10))

Intervention:

- Translabyrinthine access for resection of the vestibular schwannoma on the right side with insertion of a Contour-Advanced dummy into the cochlea on November 17, 2015

Course:

- Initial postoperative status of the facial nerve according to House-Brackmann grade VI that improved with application of a corticosteroid (dexamethasone 3x4 mg, in decreasing doses over 6 days). On the first postoperative day, already House-Beckmann grade II was achieved. Normalization of the function of the facial nerve until the first outpatient follow-up appointment on December 2, 2015.
- Hearing: right-sided deafness
- Vestibular function: increasing central compensation observed in the follow-up examinations on February 26, 2016, and June 6, 2016.

##### Stage 3

**Functional hearing ability**

**Case #6, female, age: 35 years**

History:

- Lumbar puncture in the context of neuro-borreliosis diagnostics.
- Afterwards vertigo was observed, 2 days later sudden hearing loss and tinnitus that persisted longer so that MRI of the skull was performed.
- In the further course, numbness of the left side of the face was described, mainly in the innervation area of the N. V/2-3

Clinical findings:

- Whispering was well heard in the left ear.
- Audiometry revealed class A hearing ability.
- Tendency to turn to the left in the Unterberger test.
- Stable Romberg’s test.
- Hypoesthesia in N. V/2-3 on the left.

**Figure 11:**
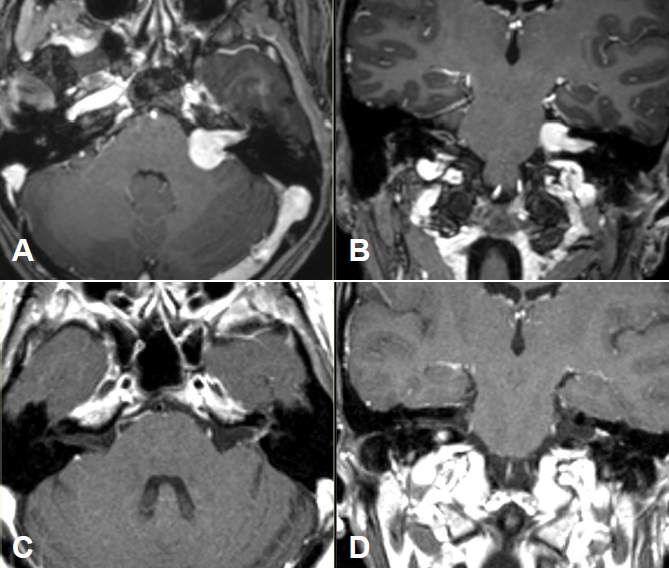
Preoperative MRI, T1-w + CM; A transversal; B. Postoperative MRI, T1-w + CM; C transversal, D coronal.

MRI:

- Vestibular schwannoma of stage T3 on the left side. The tumor grows to the fundus of the internal auditory meatus. Its cranial part touches the trigeminal nerve at the brainstem on the left (Figure 11 (Fig. 11))

Intervention:

- The patient did not wish observational strategy but primary surgery.
- Complete microsurgical tumor resection with preservation of the facial nerve and the cochlear nerve via a retrosigmoid (lateral-suboccipital) access.

Course:

- Initially, balance problems when walking fast or in the dark.
- Temporary neck pains and frontal headache, especially under stress.
- In cases of stress, pains in the area of both ears
- Left-sided hearing: sometimes hyeracusis, then phone calls with use of the left ear are uncomfortable
- Audiometry: –10 dB in the speech field in both ears.
- Speech discrimination: nearly identical in both ears
- Facial nerve: House-Brackmann grade I
- No impairment in daily life and in the job
- No recurrence over 3 years after surgery

**Functional hearing ability**

**Case #7, male, age: 52 years**

History:

- After left-sided sudden hearing loss, a vestibular schwannoma was diagnosed.
- In the context of follow-up examinations, the patient complained only about unimportant tinnitus on the left side for 5 years.
- When the tumor had significantly grown, he decided for surgery.
- Clinical findings:
- Hypoacusis in the frequencies above 2 kHz on the left to –50 dB, speech field was nearly not touched.
- Clinical balance tests were inconspicuous.

**Figure 12:**
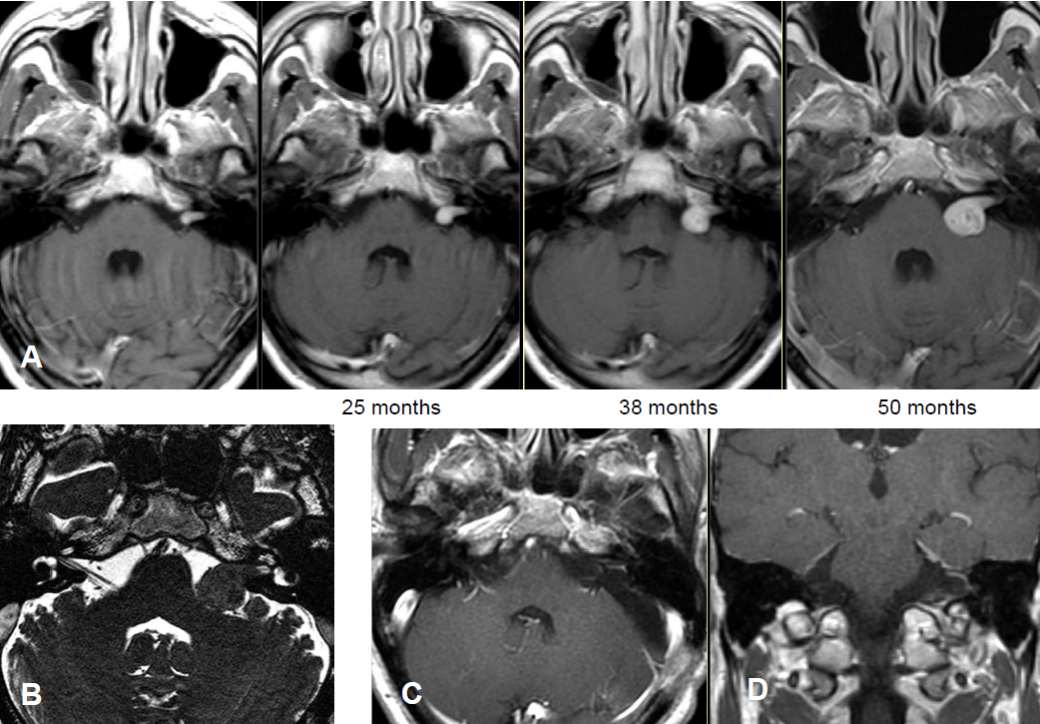
Upper row: MRI follow-up over more than 4 years; A T1-w + CM axial; lower roe: B T2 (FIESTA) transversal before surgery; T1-w + CM; C axial and D coronal postoperative.

MRI:

- Course of a primary intra-/extracanalicular vestibular schwannoma under observation until stage T3 was reached (Figure 12 (Fig. 12)).

Intervention:

- Lateral-suboccipital craniotomy on the left and microsurgically complete tumor resection with preservation of the BAEP and the facial nerve.

Course:

- Directly after surgery, slight postural instability with undirected tendency to fall when walking faster.
- Complete regression after 6 months.
- Romberg’s test was stable, Unterberger test without tendency to rotate.
- Audiometry: after one year, increased hypoacusis over all frequencies around 10–20 dB.
- In the speech field, hearing loss around 40 dB on the left.
- Inconspicuous paresis of the facial nerve.
- The tinnitus had first disappeared, later it re-appeared, but more rarely.
- Pressure sensation and numbness in the area of the left ear.

**No functional hearing ability**

**Case #8, female, age: 54 years**

History:

- 15 months before first presentation, tinnitus occurred as right-sided noise, 3 months later, also hearing impairment was noticed on the right side.
- Infusion therapy did not improve the complaints.
- MRI demonstrates a stage T3 lesion.
- Vertigo with retropulsion 6 months before first presentation, later slightly improved.

**Figure 13:**
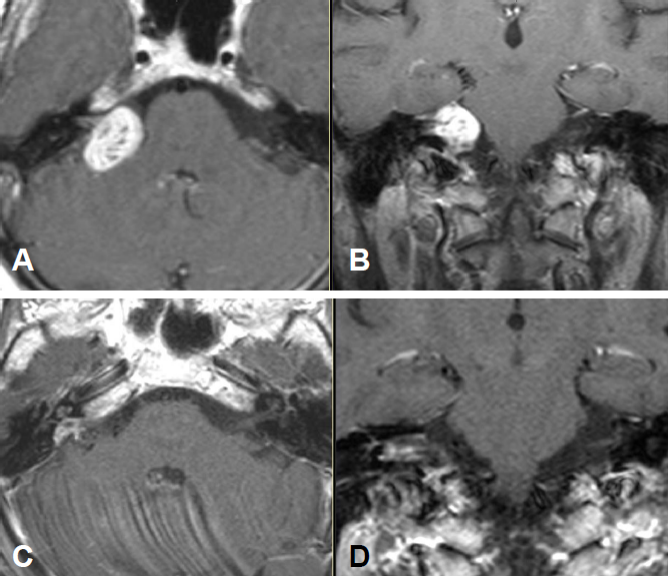
Preoperative MRI, T1-w + CM; A transversal; B coronal. The tumor reaches the fundus of the internal auditory meatus and the brainstem. MRI 4 months after surgery, T1-w + CM; C transversal; D coronal.

Clinical findings:

- Functional deafness in the right ear.
- No paresis of the facial nerve (Figure 13 (Fig. 13)).

Intervention:

- Lateral-suboccipital craniectomy of the right side and complete tumor resection by microsurgery with preservation of the facial nerve.

Course**:**

- Directly after surgery, sudden hearing loss of the contralateral side (left) with only slow recovery due to infusions and dexamethasone.
- Persisting vertigo and tinnitus.
- Romberg’s test with instability and tendency to fall until 12 months after surgery, then improvement was observed.
- The patient was more impaired by the right-sided hearing loss, especially in noisy environment.
- Apart from that, she felt well, no paresis of the facial nerve.
- 6 months after surgery, BiCROS device was applied, of which the patient was not satisfied.
- Because of unclear electrophysiological findings and negative promontory test, she decided against cochlear implantation.
- Finally, she was relevantly more satisfied after implantation of a bone anchored hearing prosthesis (BAHA).

##### Stage 4a

**Functional hearing ability**

**Case #9, female, age: 41 years**

History:

- 10 years prior to first presentation, already sudden hearing loss on the right with complete recovery due to infusion therapy.
- Because of new right-sided sudden hearing loss and whistling tinnitus, MRI diagnostics and identification of a vestibular schwannoma on the right side.
- Nearly no vertigo (dance teacher!)

Clinical findings:

- Nystagmus to the right when looking to the left.
- Instability in standing and walking tests.
- In the Unterberg test, tendency to rotate to the right.
- Subjectively low-grade hypoacusis on the right.
- Vestibular test: caloric hypoexcitability on the right side.

**Figure 14:**
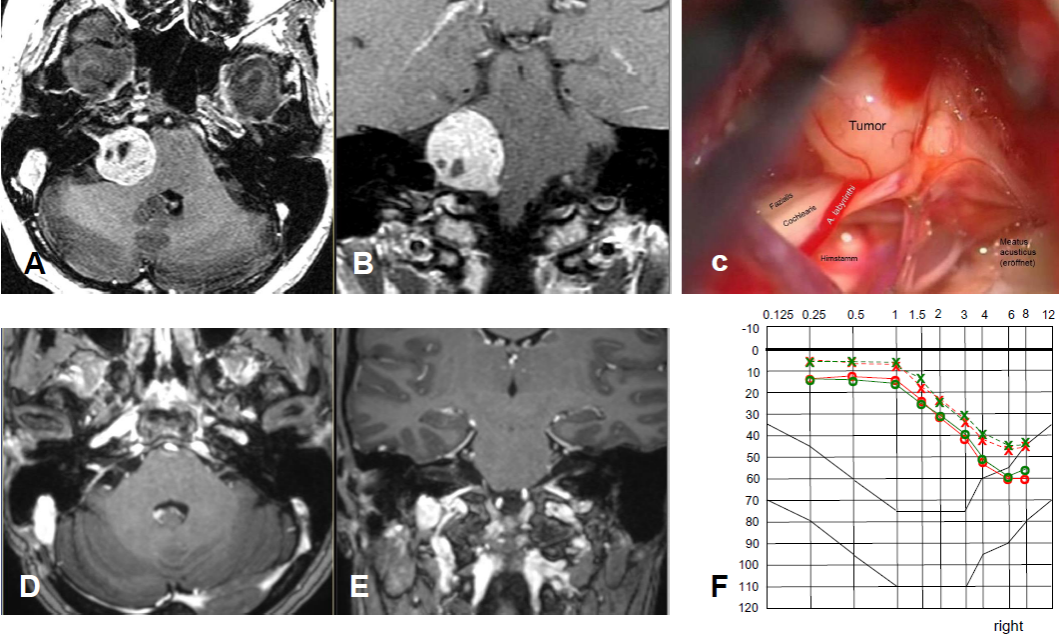
Upper row: preoperative MRI, T1-w + CM; A transversal; B coronal; C surgery site before complete resection of a residual tumor. Facial and cochlear nerves are less adherent, the labyrinthine artery is well seen. Lower row: postoperative MRI, T1-w FS + CM; D transversal; E coronal. Preoperative (green) and postoperative (red) tone audiogram. The linear contrast enhancement at the posterior wall of the right internal auditory meatus and extrameatal meningeal is a consequence of the fistula covering and not a hint to residual tumor.

MRI:

- Vestibular schwannoma on the right with brainstem compression without hydrocephalus (Figure 14 (Fig. 14)).

Intervention:

- Complete tumor resection by microsurgery with preservation of the BAEP and the facial nerve via a retrosigmoid (lateral-suboccipital) access.
- Intraoperative improvement of the acoustically evoked brainstem potentials.

Course:

- Postoperatively, first only vertigo was observed.
- 8 days after surgery, rhinoliquorrhea was found, persisting after removal of a lumbar drainage.
- 14 days postoperatively: suboccipital re-craniotomy and sealing of a fistula at the right-sided internal auditory meatus.
- Then still headache and facial pain for 2 days, especially around the right eye, then rapid improvement.
- Immediately after surgery, House-Brackmann grade II of the facial nerve, after 3 months grade I.
- Vertigo had completely disappeared after 1 year.
- Hearing ability class A as preoperatively.
- No tumor recurrence 8 years after surgery.

**Functional hearing ability**

**Case #10, male, age: 49 years**

History:

- Increasing hearing loss for 6–7 years on the left side, in the course also pressure sensation in the ear and tinnitus (whistling sound).
- No vertigo.
- Audiometry: functional hearing loss (left side), class D.

**Figure 15:**
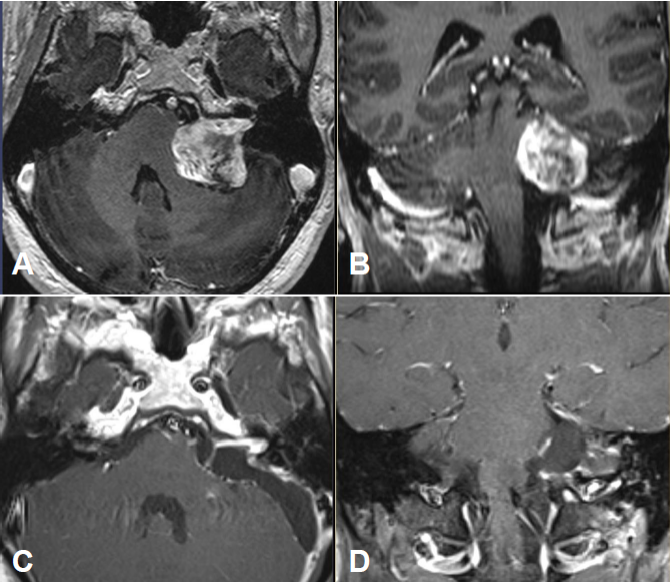
Preoperative MRI, T1-w + CM; A transversal; B coronal. 2 years after surgery, MRI T1-w + CM; C transversal; D coronal; liquor pad in the surgical pathway, linear homogeneous contrast enhancement in the left internal auditory meatus corresponding to the residual tumor.

MRI:

- Large vestibular schwannoma with growth into the fundus of the internal auditory meatus and significant brainstem compression (Figure 15 (Fig. 15)).

Intervention:

- Lateral suboccipital craniotomy and subtotal tumor resection by microsurgery with leaving a narrow tumor edge at the internal auditory meatus.
- Functional preservation of the facial nerve.

Course:

- CCT after surgery demonstrates small parenchymal bleeding in the left cerebellar hemisphere (AICA supply area) as well as blood deposits at the tentorium and the falx.
- Clinically, no cerebellar symptoms.
- No vertigo.
- Undisturbed balance.
- Clinical paresis of the facial nerve, House-Brackmann grade II on the left, after 3 months, grade I.
- Tinnitus had disappeared.
- No recurrences after 2 years.

##### Stage 4b

**Functional hearing ability**

**Case #11, female, age: 34 years**

History:

- 6 months before first presentation, bilateral hearing loss after common cold.
- Recovery of the right side to normal level; on the left side, persisting higher-grade hearing loss.
- No relevant vertigo.

Clinical findings:

- Instable Romberg’s test.
- Unterberg test with rotation to the right.
- Left-sided hypoacusis
- No other deficits.
- Bilateral papilledema (vision of the left eye: 0.2; already poor in childhood).
- Directly before surgery nausea and vomiting.

**Figure 16:**
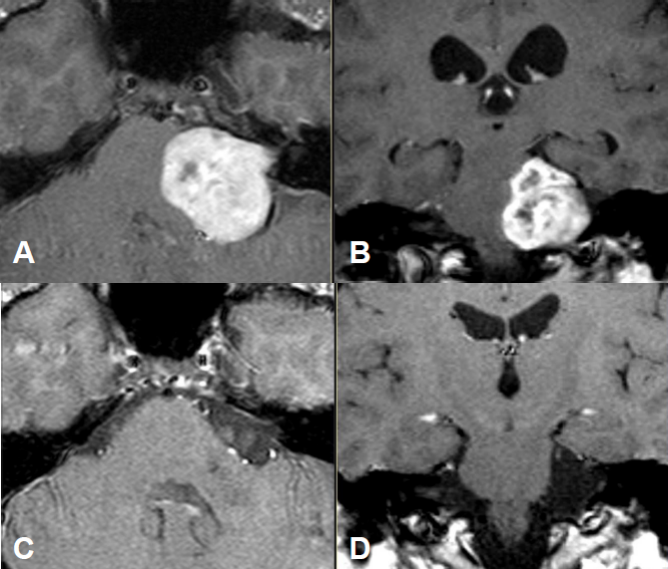
Preoperative MRI, T1-w + CM; A transversal; B coronal. 1 year after surgery, MRI, T1-w + CM; C transversal; D coronal; gliosis zone in the left cerebellar peduncle as consequence of the compression by the tumor. The hydrocephalus regressed, the ventricular system is again normally wide.

MRI:

- Stage T4b vestibular schwannoma on the left side with compression of the aqueduct and obstructive hydrocephalus.
- Meningioma in the left olfactory area (not depicted) as additional finding (Figure 16 (Fig. 16)).

Intervention:

- External ventricular drainage and microsurgical resection of the vestibular schwannoma via a retrosigmoid access on the left side.

Course:

- Immediately postoperative paresis of the facial nerve, House-Brackmann grade V, on the left.
- Left-sided anacusis.
- Further neurological status was inconspicuous.
- 1 year after surgery, facial nerve, House-Brackmann grade II, afterwards the patient did not present again for clinical follow-up.
- MRI performed 2 years after surgery, did not show tumor recurrence.
- No hydrocephalus (shunt was not necessary).

##### Stage 4a with complicated treatment course

**Recurrence after surgery and radiosurgery,** **hydrocephalus**

**Case #12, female, age: 65 years**

History:

- Ear noise on the left 1 year before first presentation, improved with pharmacotherapy, then again increasing.
- Diagnostics by means of MRI after observation of increasing numbness of the whole left side of the face. Further increasing painful dysesthesia in this area.
- Left-sided hearing loss, phone calls using the left ear were no longer possible.
- Arterial hypertonia.

**Figure 17:**
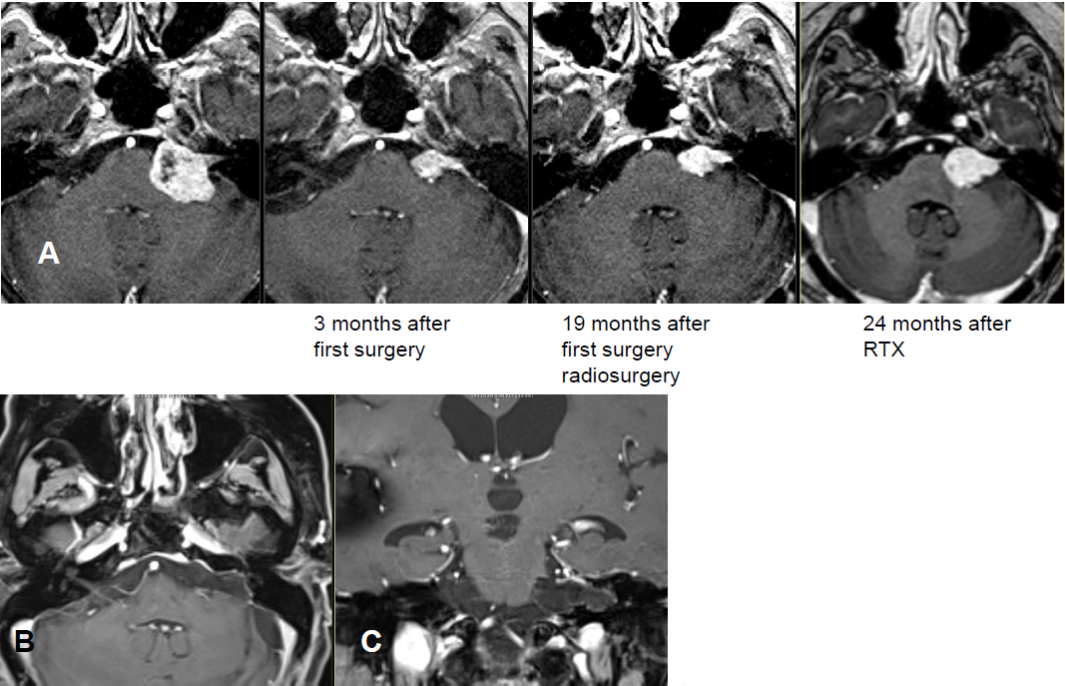
Upper row: preoperative MRI (T1-w + CM; A transversal) and follow-up MRI. 3 months after the 1^st^ surgery, there is still a larger residual tumor in situ. After 19 months is has slightly increased, also 2 years after irradiation, the tumor is progressive, the brainstem is compressed. Lower row: MRI 6 months after the 2^nd^ resection. T1-w + contrast agent application; B transversal; C coronal.

MRI:

- Vestibular schwannoma on the left side, stage T3 (Figure 17 (Fig. 17)).
- Clinical findings:
- Hypoesthesia in N. V/1-3 on the left side. Hypoacusis in the left ear.
- Deviation to the right in the Unterberg test.
- Laboratory chemical parameter: renal insufficiency grade II

1. Intervention:

- Lateral suboccipital craniotomy and partial microsurgical resection of a highly vascularized tumor with preservation of the facial nerve and the cochlear nerve.

Course:

- Postoperatively, relevant headache symptoms, computed tomography did not reveal hydrocephalus.
- Significant improvement with symptomatic therapy.
- Facial nerve: House-Brackmann grade I.
- Tinnitus and hearing like before surgery.
- Stable gait.
- 1.5 years after surgery, persisting headaches and post-therapeutic facial pain. Improvement with application of carbamazepine and gabapentin.
- MRI of the skull revealed a minimal growth of the residual tumor over 6 months.

2. Intervention:

- Unilateral irradiation in CyberKnife.
- First MRI follow-up revealed a stable situation. 2 years later, increasing memory disorders with massive gait instability and persisting headaches. Detection of hydrocephalus and implantation of a ventriculo-peritoneal shunt system at an opening pressure of 21 cm water column.
- Because of staphylococcus infection, removal and re-implantation of an adjustable shunt system (pro GAV II, Mietke-Aesculap).
- At rest, recurrent paresis of the buccal branch of the left facial nerve were observed.
- 2 months later, again increasing gait instability (fall in the shower) and dizziness, in particular with massive episodes when the patient got up from a lying position. She had difficulties to walk at all because of the vertigo.
- Less headaches, only in the morning and evening.
- MRI of the skull did not show an increased ventricular width, but a clear growth of the residual tumor in the left cerebellopontine angle.
- Stop of the use of carbamazepine and gabapentin, then slight improvement of the vertigo symptoms.
- Meanwhile functional anacusis on the left side and increased paresis of the buccal branch of the facial nerve (House-Brackmann grade II).

3. Intervention:

- Lateral suboccipital re-craniectomy on the left side and subtotal microsurgical tumor resection with preservation of the affected facial, glossopharyngeal, abducens, and trigeminal nerves.
- Between the meatus and nearly in the center of the cerebellopontine cistern, a very thin tumor layer has to remain on the facial nerve because further resection would not have allowed functional preservation of the nerve.

Histology:

- Schwannomas with significant regressive changes, bleedings of different times as well as slight overexpression of p53 and p21.
- No anaplasia, no malignancy.

Further course:

- Postoperative paresis of the facial nerve unchanged with grade 2 on the left side. Still slight walking and standing instability and slight vertigo.
- In the further course, again increasing painful dysesthesia in the supply area of the left trigeminal nerve (VAS 7-8/10 for some hours per day). Recurrent reddening of the left eye. After administration of Lyrica 75 mg and Amitriptyline 50 mg 0-0-1, improvement of the painful dysesthesia.
- MRI performed 6 months after surgery, did not reveal tumor recurrence.

##### Cystic vestibular schwannoma, stage 4a

**Functional hearing loss**

**Case #13, male, age: 43 years**

History:

- Tinnitus rapidly progressing over 3 months and increasing hearing loss in the right ear so that finally the right ear could no longer be used for phone calls.
- In addition, progressive gait instability.

Clinical findings:

- Romberg’s test was insecure with tendency to fall. Unterberg test could not be performed because of tendency to fall.
- High-grade hearing loss on the right.
- Audiometric inner ear hearing loss, on average >–60 dB in the speech field, right-sided.

**Figure 18:**
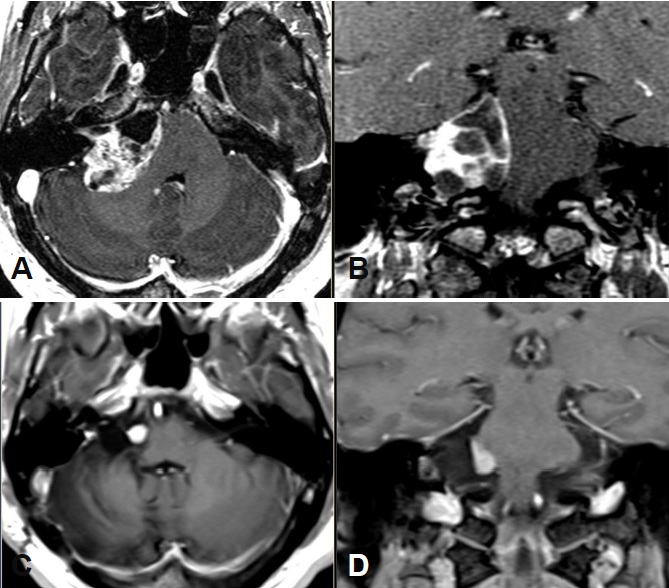
Preoperative MRI, T1-w + CM; A transversal; B coronal. MRI 9 years after surgery, T1-w + CM; C transversal; D coronal. Intensive homogeneous contrast enhancement of the residual tumor.

**Figure 19:**
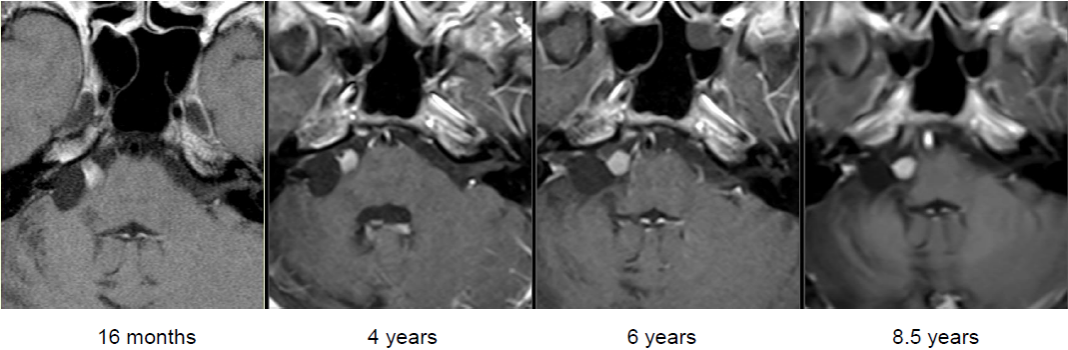
Up to now, the postoperative MRI follow-up (T1-w + CM, axial) did not show any growth of the residual tumor.

MRI:

- Large cystic tumor filling the cerebellopontine angle with brainstem compression and advancing into the right internal auditory meatus (Figure 18 (Fig. 18) and Figure 19 (Fig. 19)).

Intervention:

- Lateral suboccipital craniotomy and microsurgical partial resection of a highly vascularized tumor that relevantly split up the facial nerve.

Course:

- Immediately after surgery, right-sided paresis of the facial nerve (House-Brackmann grade II).
- Massively increasing vertigo and in particular image fusion disorder due to gaze-evoked nystagmus to the left. It was caused by a circulatory disorder in the area of the inferior cerebellar peduncle and the cerebellar hemisphere.
- Additional complication because of gout attack in the right ankle.
- 6 months after surgery only slight tinnitus perceived as weak noise.
- Improvement of the gait instability and still disturbed image fusion when looking straight forward as well as diplopia and blurred vision when looking to the right without visible weakness of the abducens nerve.
- Rotatory nystagmus when looking to the right. Slightly delayed diadochokinesia on the right side.
- No longer relevant vertigo. No longer paresis of the facial nerve (House-Brackmann grade I).
- Romberg’s test still unstable, but compensated.
- 2 years after surgery, the patient is again full-time working. Romberg’s test is stable, Unterberger test is insecure without tendency to fall and without rotating tendency.
- MRI did not reveal growth of the residual tumor for 8 years.

### 9.2 Radiosurgery (RS) and stereotactic radiotherapy (SRT)

#### 9.2.1 Typical courses after RS/SRT

The term of “radiosurgery” was used by the Swedish neurosurgeon Leksell for a method developed by him in the 1950ies. It was a method of high-precision convergence irradiation with a high dose, that is applied once. The patient's head first had to be fixed in a stereotaxy frame (about 0.3 mm precision) similar to stereotactic surgeries. Since the 1980ies, the stereotactic positioning of the patient is applied with a special, less precise mask fixation (±1-2 mm precision) than SRT for the fractioning that is used in radiotherapy. From 2000 to 2012, we performed RS and SRT with the dedicated linear accelerator Novalis (BrainLAB) - because of the longer follow-ups, the case examples were mostly recruited from the population treated with the Novalis. Since October 2012, we work with the robot-assisted radiosurgery system CyberKnife (Accuray) that allows the radiosurgical precision of about 0.3 mm due to permanent image control even without frame with mask positioning. Instead of SRT, the treatment of larger tumors can be limited to 3 single irradiations, the so-called multisession radiosurgery (msRS).

First some examples of mainly typical courses will be presented (cases 1–8), then particular, atypical courses will be described that also include severe single cases (cases 9–14).

##### Stage 1

**Functional hearing ability**

**Case #1, female, age: 41 years**

History:

- 3 years before initial presentation, left-sided sudden hearing loss without remaining hearing impairment. Tinnitus in the left ear as noise, not disturbing. In the course, sometimes vertigo and imbalance. In the MRI follow-up of 09/2012 and 04/2013, significant tumor growth of max. 4 mm to 8x6 mm into the left meatus.

Clinical findings:

- Unterberger test instable with undirected tendency to fall.
- The audiogram revealed inner ear hearing loss of 55 dB on average in the speech field.

**Figure 20:**
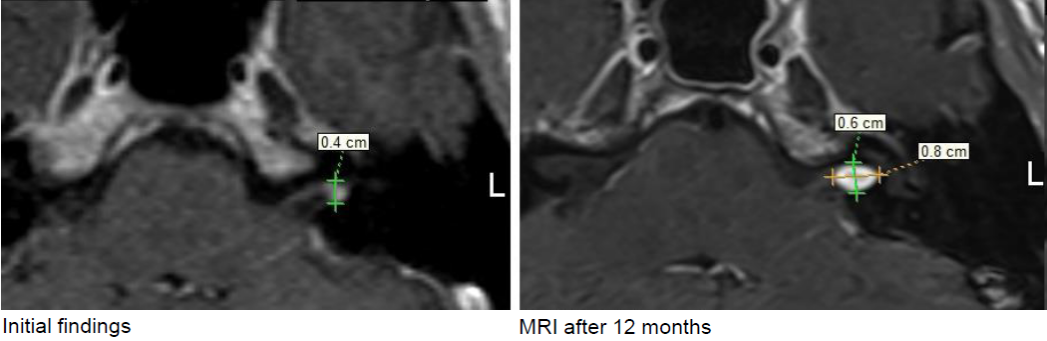
Intrameatal vestibular schwannoma, growing after 7 months from 4x4 mm to 8x6 mm; T1-w + CM, axial; A initial findings; B MRI control after 7 months.

MRI:

- Figure 20 (Fig. 20).

Intervention:

- Radiosurgery (CyberKnife, margin dose of 12 Gy at 69% isodose).

**Figure 21:**
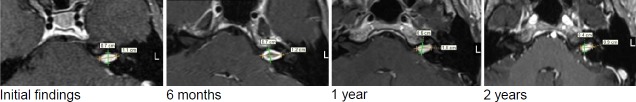
The MRI follow-up control (T1-w + CM, axial) after therapy first show a minimal growth with cystic transformation of the tumor and in the further course a reduced volume.

Course:

- 6 months after the intervention, the hearing ability was unchanged.
- MRI revealed the typical reaction with central contrast agent reduction and relevant contrast enhancement in the edges showing a transiently rather small tumor. In the further course until 12/2015, regression of the reactive changes and regredient tumor size with still well-being and good hearing ability over meanwhile 2.5 years (Figure 21 (Fig. 21)).

In the following case examples, the sides in the MRI series are mirrored because of the applied stereotactic planning software. Since the assessment of the course is most relevant and no information gets lost due to this exchange of the sides, the images are not displayed in a mirrored way.

##### Stage 2

**Functional hearing loss**

**Case #2, male, age: 68 years**

History:

- 4 years before first presentation, surgery of a right-sided vestibular schwannoma (retrosigmoid access).
- Tinnitus and occasional vertigo.
- Alternating hypo- and dysesthesia of the right side of the face.

Clinical findings:

- Anacusis in the right ear.
- Labyrinth failure on the right.
- Unterberger test compensated.
- Hypo- and dysesthesia in the supply area of the 2^nd^ trigeminal ramus (right-sided).
- Paresis of the facial nerve (House-Brackmann grade III (right side).

MRI:

- Intra-/extrameatal tumor recurrence (16x9 mm), progressing compared to the previous examination one year before.

Intervention:

- Radiosurgery (linear accelerator Novalis, Brainlab, margin dose of 12 Gy at 80% isodose).

**Figure 22:**
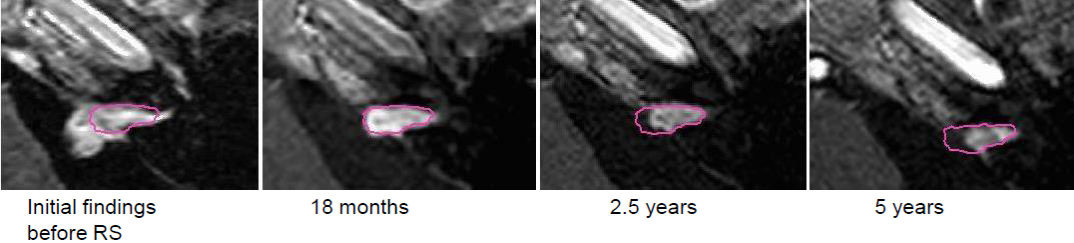
The MRI follow-up examinations (T1-w + CM) show progressive tumor shrinking. The pink line corresponds to the tumor size 18 months after radiosurgery.

Course:

- 7 months after intervention, the patient reported about an improvement of the hypo- and dysesthesia of the right side of the face, the other symptoms were unchanged.
- In the further course, gradual improvement of the paresis of the facial nerve and significant tumor regression confirmed by MRI (Figure 22 (Fig. 22)).

**Functional hearing loss**

Case #3, female, age: 64 years

History:

- Right-sided tinnitus for 3 years and balance disorders that have increased gradually.
- Hearing loss in the right ear was first audiometrically stable with high frequency loss on the right, now increasing up to 50 dB.

Clinical findings:

- Romberg’s test instable.
- Unterberger test with severe, undirected tendency to fall, dysdiadochokinesis on the left.
- Hypoacusis on the left side.

MRI:

- In the course of the disease, progressive right-sided VS intra- and extrameatal corresponding to stage 2 (max. diameter of 13 mm, extrameatal 7 mm).

Intervention:

- Radiosurgery (linear accelerator Novalis, Brainlab, margin dose of 12 Gy at 80% isodose).

**Figure 23:**
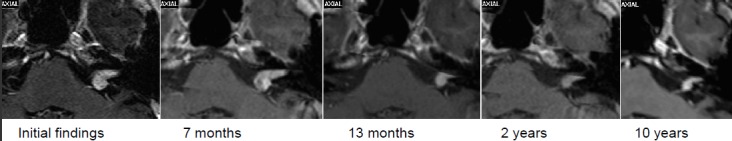
The MRI follow-up examinations (T1-w + CM) show progressive shrinking of the tumor at 7 months after RS onwards.

Course:

- 6 months after intervention slightly increasing hearing loss in the right ear.
- Disturbed balance did not increase.
- In the further course, increasing right-sided hearing loss with steep decrease to 70 dB.
- The vestibular nerve can be stimulated on both sides.
- Clear tumor regression in the MRI follow-up.
- 4 years after radiosurgery, nearly stable MRI findings and stable clinical course (Figure 23 (Fig. 23)).

**Functional hearing ability**

**Case #4, male, age: 60 years**

History:

- For about 1 year, hearing deterioration on the left side observed during phone calls.
- Increasing tinnitus and pressure sensation in the left ear, no vertigo.

Clinical findings:

- Audiometry revealed left-sided sensory hearing loss up to 80 dB in the high frequencies.

MRI:

- Vestibular schwannoma on the left side, in the longitudinal axis 15 mm intra- and extrameatal nearly reaching the brainstem.
- Progressive growth in the further course.

Intervention:

- Radiosurgery (linear accelerator Novalis, Brainlab, margin dose of 12 Gy at 80% isodose).

**Figure 24:**
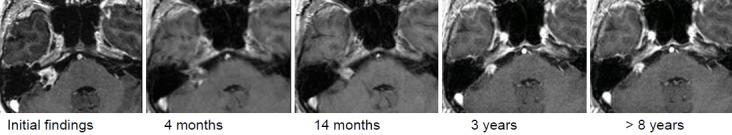
The MRI follow-up examinations (T1-w + CM) show progressive tumor shrinking. Already prior to therapy, the tumor is inhomogeneous, after RS further regressive central contrast enhancement. In the follow-up, regression of tumor size up to 3 years after RS, then constant tumor size over >8 years.

Course:

- 4 months after intervention, clinically and ENT-specifically no deterioration.
- In the MRI, typical central reduction of contrast enhancement.
- In the further course, regression of the reactive changes and regressive tumor size in the MRI follow-up until 3 years after radiosurgery, then stable condition.
- Slightly progressive hearing loss in the deep and middle frequencies of 50–90 dB, constant over >8 years (Figure 24 (Fig. 24)).

##### Stage 3

**Functional hearing ability**

**Case #5, female, age: 58 years**

History:

- Left-sided hearing loss with functional remaining hearing ability (–25 dB in the speech-field).
- Rotational vertigo and balance disorder.
- Hypo- and dysesthesia of the left side of the face.
- MRI follow-up showing progressive VS on the left, now with brainstem contact.

Intervention:

- Radiosurgery (linear accelerator Novalis, Brainlab, margin dose of 12 Gy at 80% isodose).

**Figure 25:**
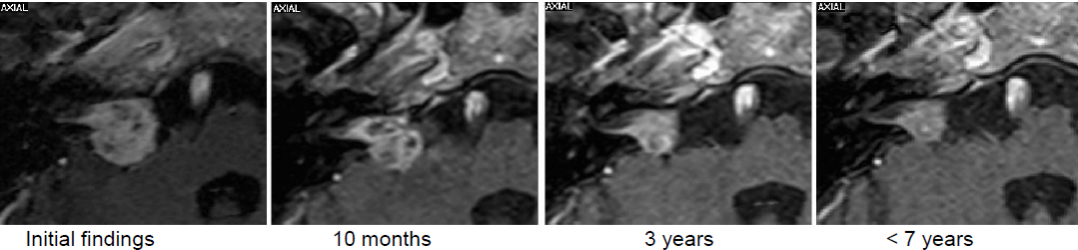
The MRI follow-up examinations (T1-w + CM) show progressive tumor shrinking.

Course:

- 6 months after intervention, unchanged symptoms.
- Hearing ability after >7 years on the left side with pancochlear inner ear hearing loss to 85 dB at 6 kHz, in the speech field about 20 dB poorer than initial audiometry.
- Clinically, further dysesthesia in the area of the left corner of the mouth and the tongue (Figure 25 (Fig. 25)).

**Functional hearing ability**

**Case #6, female, age: 24 years**

**Figure 26:**
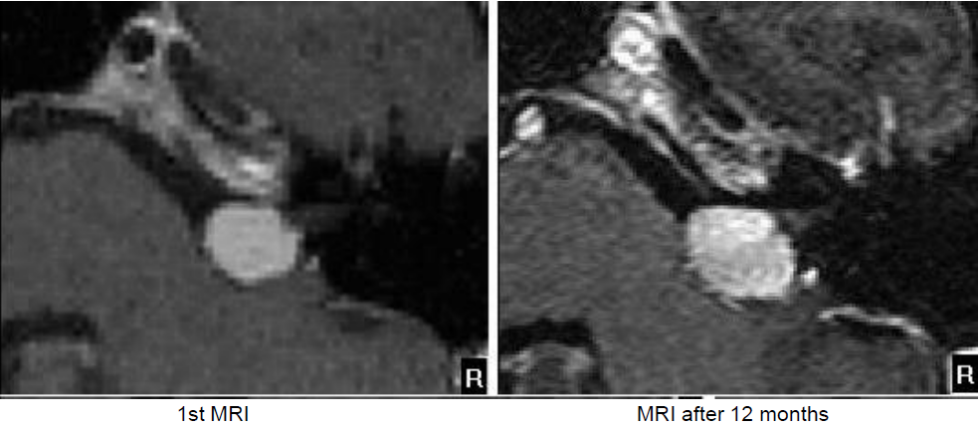
The MRI follow-up examinations (T1-w + CM) show an increased tumor after 1 year.

History:

- After right-sided sudden hearing loss, MRI confirmed vestibular schwannoma on the right side.
- Then wait-and-see strategy due to complaint-free clinical findings and bilaterally good hearing ability.
- The MRI follow-up showed clear tumor progression of the tumor volume from 0.86 cm³ to 1.45 cm³ (Figure 26 (Fig. 26)).

Clinical findings:

- Romberg’s test stable.
- Unterberger test with mild insecurity.

Intervention:

- The patient refused the surgery recommended by the interdisciplinary team.
- Radiosurgery (linear accelerator Novalis, Brainlab).

**Figure 27:**
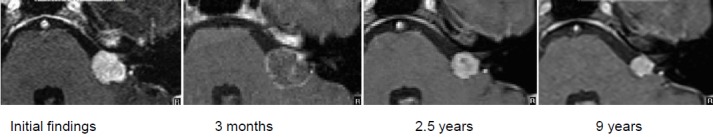
The MRI follow-up examinations (T1-w + CM) show first a clear growth of the tumor with low marginal contrast enhancement 3 months after RS, in the further course, shrinking of the tumor and again homogeneous contrast agent application.

Course:

- Due to vertigo and painful dysesthesia on the right side of the face, already 3 months after intervention the first control MRI was performed. It revealed an extraordinarily severe reaction with central reduction of contrast agent and increased volume.
- In the further course, nearly complete regression of the complaints.
- 9 years after the intervention, still good hearing ability in both ears (Figure 27 (Fig. 27)).

##### Stage 4a

**Functional hearing loss**

**Case #7, female, age: 67 years**

History:

- For many years, right-sided hearing loss, finally no useable hearing ability on the right side.
- Tinnitus.
- Occasionally vertigo and balance disorders.

Clinical findings

- Anacusis in the right ear.
- Hypostimulation of the: right labyrinth.

MRI:

- The MRI dated 08/2004 showed a typical VS on the right side with compression of the brainstem (stage 4a), progressive in the further course.

Intervention:

- The patient refused the surgery recommended by the interdisciplinary team.
- Fractionated stereotactic radiotherapy (SRT) with 27x2 Gy (Novalis, Brainlab) at a tumor volume of 6.5 cm³.

**Figure 28:**
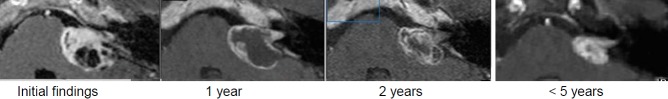
The MRI follow-up examinations (T1-w + CM) first show a nearly constant tumor size with only low marginal contrast enhancement (1 year after RS), in the following years, progressive tumor shrinking with increasing contrast enhancement.

Course:

- 6 months after irradiation, the patient reported about unchanged compensated gait insecurity and occasional dysesthesia on the right side of the face that disappeared in the further course.
- The MRI control examinations first showed a central hypointensity, then increasing tumor regression to 1.8 cm³ tumor volume.
- Last follow-up examination in 02/2014 revealed unchanged findings (9 years after SRT) (Figure 28 (Fig. 28)).

**Functional hearing loss**

**Case #8, male, age: 47 years**

History:

- Condition after surgery of vestibular schwannoma of the right side 7 and again 4 years before. Now again recurrence with stage 4a.
- Tinnitus and balance disorders.

Clinical findings:

- Anacusis and facial hypoesthesia of the right side.
- No paresis of the facial nerve.

MRI:

- Tumor recurrence on the right side, stage 4a.
- Tumor volume of 9.6 cm³.

Intervention:

- Fractionated stereotactic radiotherapy (SRT) with 27x2 Gy (Novalis, Brainlab).

**Figure 29:**
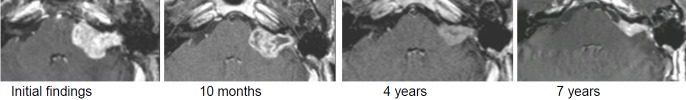
10 months after SRT, the MRI follow-up controls (T1-w + CM) show a shrinking of the tumor with only low marginal contrast enhancement, in the following years, progressive tumor shrinking with increasing contrast enhancement.

Course:

- 7 months after irradiation, the patient reported about an increased hypoesthesia on the right side of the face. Labyrinth failure and anacusis on the right side were unchanged.
- The first 2 MRI control examinations in 2003 showed the typical, central hypointensity of the contrast agent.
- In the further course, relevant tumor shrinking to 1.1 cm³ in 09/2011 with clinically stable findings (Figure 29 (Fig. 29)).

#### 9.2.2 Untypical courses after radiosurgery or stereotactic radiotherapy

##### Stage 3

**Functional hearing ability**

**Case #9, male, age: 44 years**

History:

- Increasing hearing loss on the left side to 50 dB.
- Tinnitus.

MRI:

- The MRI showed a progressive vestibular schwannoma on the left side of stage 3, mainly extrameatal location.

Intervention:

- The patient refused the recommended surgery
- Fractionated stereotactic radiotherapy (SRT) with 27x2 Gy (Novalis, Brainlab).

**Figure 30:**
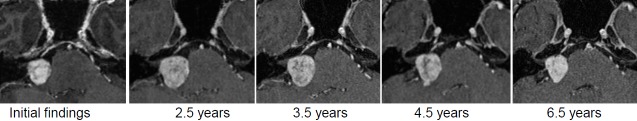
In the first years, the MRI follow-up examinations (T1-w + CM) show a growing tumor with nearly unchanged inhomogeneous contrast enhancement, only after 3.5 years, the tumor is shrinking.

Course:

- In the course, first increasing tumor volume was observed from 3.8 cm³ to 7.3 cm³ after 2.5 years. The recommended surgery was refused; stable clinical findings and constant hearing loss.
- In the further course, regression of the tumor.
- Clinically improved hearing loss (–40 dB in the speech field) and occasional balance disorders.
- The patient is fully able to work (Figure 30 (Fig. 30)).

##### Stage 2

**Functional hearing ability**

**Case #10, male, age: 41 years**

History:

- Increasing hearing deterioration on the left side to 50 dB.
- Increasing tinnitus.

MRI:

- Progression of the vestibular schwannoma on the left side, stage 2.

Intervention:

- The patient refused the recommended surgery
- Radiosurgery (linear accelerator, Novalis, Brainlab; margin dose of 12 Gy at 80% isodose).

**Figure 31:**
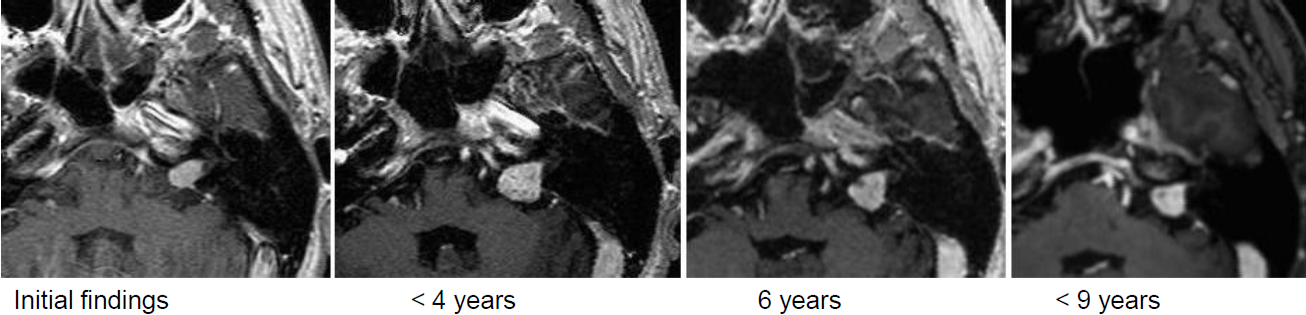
In the first years, the MRI follow-up control (T1-w + CM + contrast agent application) show a growing tumor with nearly unchanged contrast enhancement, only after 6 years, the tumor volume decreases.

Course:

- In the course, first increasing tumor volume from 0.6 cm³ to 1.5 cm³ after nearly 4 years; the recommended surgery was refused. Stable clinical findings and constant hearing loss.
- In the further course, tumor regression with unchanged clinical findings.
- The patient is fully able to work (Figure 31 (Fig. 31)).

##### Stage 2

**Functional hearing loss**

**Case #11, female, age: 75 years**

History:

- The left ear was functionally deaf.
- Alternating tinnitus.
- Unsteady gait.

MRI:

- Progressive vestibular schwannoma on the right side, stage 2.

Intervention:

- Radiosurgery (linear accelerator Novalis, Brainlab, margin dose of 12 Gy at 80% isodose).

**Figure 32:**
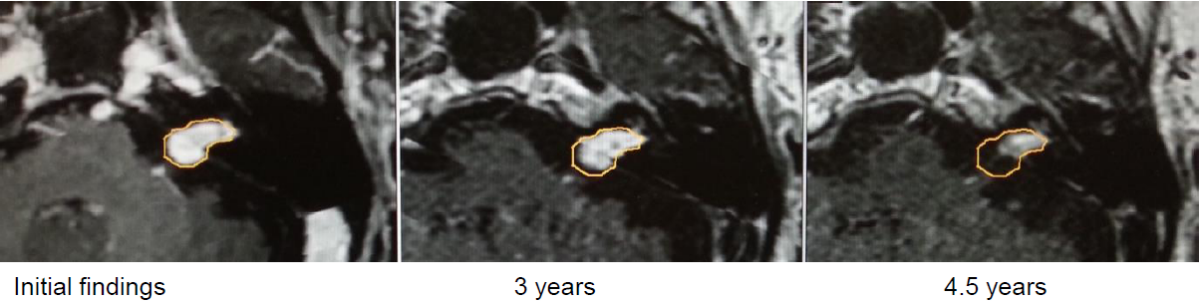
After 3 years, the MRI follow-up examinations (T1-w + CM) only show minimal size reduction, after 4.5 years, clear regression, the extrameatal part has nearly completely disappeared.

Course:

- First typical course with central hypointensity of the contrast agent after 6 months and then marginal regression until 3 years after intervention.
- 1.5 years later, only an intrameatal residual tumor was found with unchanged clinical findings (Figure 32 (Fig. 32)).

**Functional hearing loss**

**Case #12, female, age: 73 years**

History:

- Left-sided hearing loss for about 2 years.
- Increasing vertigo attacks and suddenly appearing balance disorders
- Left-sided tinnitus.
- Left-sided facial dysesthesia.

MRI:

- Left-sided vestibular schwannoma, stage 3, with a volume of 2.2 cm³.

Intervention:

- Radiosurgery (CyberKnife, Accuray, margin dose of 12 Gy, 69% isodose).

**Figure 33:**
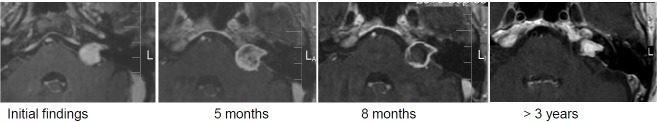
The MRI follow-up controls (T1-w + CM) show an increased size after 5 months with regressive central contrast enhancement, after 8 months only marginal contrast enhancement and constant size. After 3 years, shrinking tumor with again homogeneous and intensive contrast enhancement.

Course:

- After 5 months, the tumor volume appears larger with focal spots of absent contrast enhancement. In the next follow-up examination cystic involutions becomes evident, but the tumor size remains constant.
- Clinically stable findings.
- One year later, significant regression to a volume of 0.5 cm³.
- Subjectively neither vertigo nor balance disorders.
- Less important tinnitus (Figure 33 (Fig. 33)).

**Functional hearing loss**

**Case #13, female, age: 59 years**

History:

- Unsteady gait.
- Increasing left-sided hearing loss.

Clinical findings:

- Unterberger test: deviation to the left in blind walking.
- Inner ear hearing loss with audiometric hearing loss to 80 dB at 1.5 to 4 kHz.

MRI:

- Progressive vestibular schwannoma of the left side, stage 2 (1.3 cm³).

Intervention:

- Radiosurgery (linear accelerator Novalis, Brainlab, margin dose of 12 Gy at 80% isodose).

**Figure 34:**
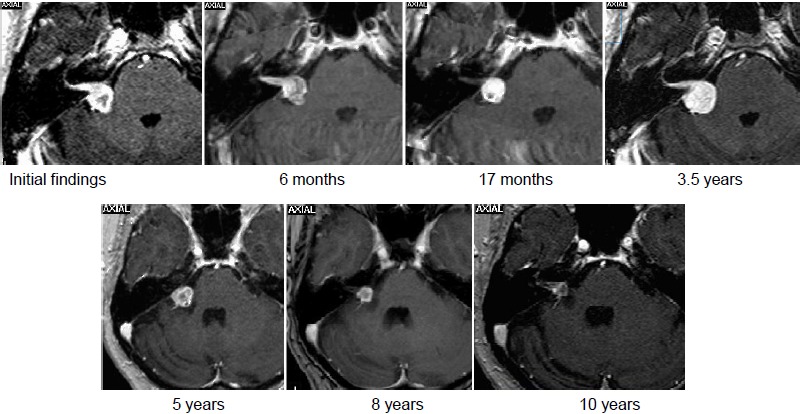
The MRI follow-up controls (T1-w + CM) show an inhomogeneous contrast enhancement of the tumor before therapy. After 6 months, minimal shrinking with slightly reduced contrast enhancement. After 17 months, again increased contrast enhancement with unchanged tumor size. After 3.5 years again tumor growth to 2.1 cm³. After second radiosurgery with 12 Gy margin dose (80%) again central contrast decrease and reduction of the tumor size to 0.12 cm³.

Course:

- Stable clinical findings with hearing aids on the left side.
- After 3.5 years, recurrent tumor growth, clinically associated with functional hearing loss and increasing balance disorders.
- The suggested surgery was refused, the second irradiation was performed with a margin dose of 12 Gy (80%).
- The clinically painful dysesthesia occurred and convulsions on the left side of the face required pharmacotherapy and local botox injections (Figure 34 (Fig. 34)).

##### Stage 4

**Functional hearing loss**

**Case #14, female, age: 63 years**

History:

- 3.5 years after resection, progressive residual tumor on the right side.
- Condition after tarsorrhaphy and neuromuscular anastomosis on the right side.
- Additional findings: diabetes mellitus and chronic renal insufficiency.

Clinical findings:

- Anacusis and complete paresis of the facial nerve on the right side.
- Significant vestibular gait disorder and hypoesthesia of the right side of the face.

MRI:

- Progressive vestibular schwannoma on the right side, stage 4, with a volume of 5.5 cm³.

Intervention:

- Revision surgery was refused.
- Fractionated stereotactic radiotherapy (SRT) with 30x1.8=54 Gy (Novalis, Brainlab).

**Figure 35:**
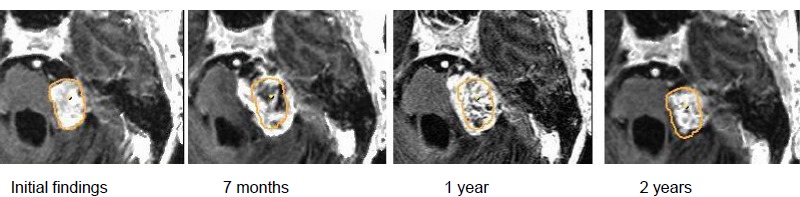
The MRI follow-up controls (T1-w + CM) show an inhomogeneous contrast enhancement of the tumor before therapy. 7 months after SRT, increased size and surrounding edema, centrally reduced contrast enhancement. After 1 year, constant size with again increased contrast enhancement. In the further course, regressing tumor volume. After 4.5 years, again tumor progress (the orange line corresponds to the tumor size at the time of 1^st^ SRT).

**Figure 36:**
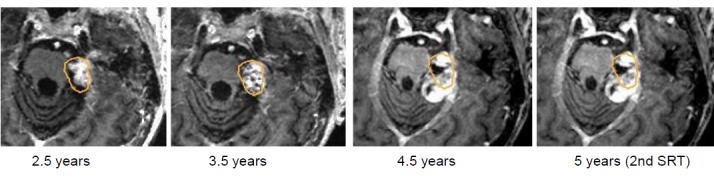
Best clinical condition and MRI findings up to 2.5 years after irradiation further marginal tumor regression. 4 years later, again significant tumor growth to 14.2 cm³. Meanwhile, the patient had to undergo dialysis because of renal failure. She strictly refused surgery. Upon her urgent wish, again SRT with 22x1.8 Gy was started that had to be interrupted after the 16th fraction because of a fall with bilateral fractures of the ischium and the pubic bones and intensive care that was necessary because of metabolic imbalance. The patient dies of multiorgan failure.

Course:

- After 7 months, frequent pain attacks of the right trigeminal nerve, other neurological findings were unchanged.
- MRI showed a severe reaction on SRT with increasing growth and surrounding edema at that time.
- In the further course, first regression of the reactive changes with clinical improvement of the balance disorders and no more pain attacks (Figure 35 (Fig. 35), Figure 36 (Fig. 36)).

### 9.3 Wait and scan

**Functional hearing ability**

**Case #1, male, age: 40 years**

History:

- In the context of diagnostics for headache in 2008, MRI confirming an intrameatal acoustic neuroma of the right side of less than 3 mm.
- Control MRI in February 2009 revealed a constant size of about 3x2 mm.
- Subjectively no vestibular disorder with temporary right-sided tinnitus and bilateral subjectively normal hearing.
- Additional findings: recurrent sinusitis, otherwise inconspicuous history.

Clinical findings:

- Normal hearing on both sides, facial nerve: House grade I, no vestibular disorders.

Intervention:

- Wait and scan.

**Figure 37:**
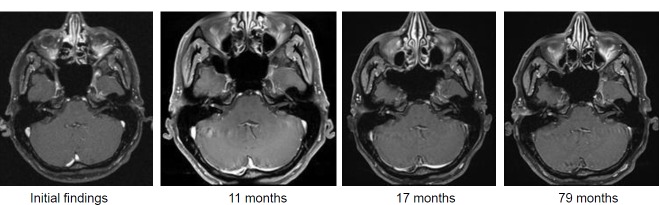
Axial MRI, T1–w + CM; constant size of a vestibular schwannoma during a follow-up of 6.5 years.

Course:

- Annual follow-up examinations in 9–12 months interval.
- MRI revealed constant tumor size of 3x2x2 mm.
- Tone and speech audiometry in the normal range.
- The balance tests showed no hint for peripheral-vestibular disorder.
- During the regular follow-up examinations, we explained and offered all therapeutic options to the patient. Because of missing clinical complaints, the patient decided for further regular tumor observation in the sense of wait-and-scan (Figure 37 (Fig. 37)).

**Functional hearing loss**

**Case #2, male, age: 73 years**

History:

- Progressive hearing loss on the right side for several years.
- Additionally, especially in the morning, slight vertigo for some months.
- Beside arterial hypertension, inconspicuous anamnesis.

Clinical findings:

- Inconspicuous status of the facial nerve as well as slight vestibular disorder in the morning.

MRI:

- Intrameatal acoustic neuroma on the right side with a size of 0.7x0.3 mm.

Intervention:

- Wait-and-scan.

**Figure 38:**
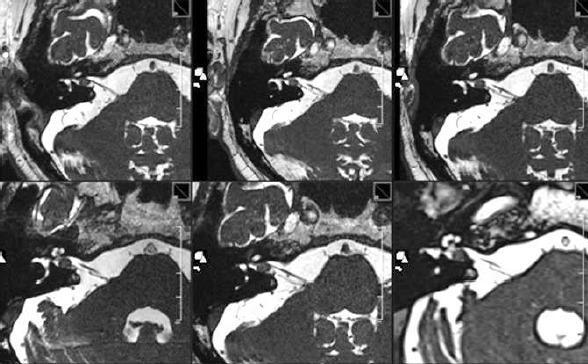
MRI 1.5 T; CISS axial; 0.8 mm slice thickness. Upper row: 5 years after diagnosis; 4 years after diagnosis; 3 years after diagnosis. Lower row: 2 years after diagnosis; 1 years after diagnosis; initial diagnosis.

Course:

- Annual follow-up examinations in 9–12 months intervals.
- Subjective deterioration of the right-sided hearing.
- First recommendation of surgical tumor resection with explanation of alternative therapeutic options by means of radiotherapy or further observation. The patient decided for clinical and MRI controls.
- One year later, again subjective deterioration of the right-sided hearing as well as slightly increased vertigo symptoms. The subjectively increased hearing loss was confirmed by tone and speech audiometry. An increase of the initial hearing loss (45%) was measured now at 65% according to Röser (1980) [36] and a reduced Freiburg speech test for polysyllables as well as for monosyllables.
- In the further course, balance tests did not reveal any hint for peripheral vestibular disorders.
- Over the time of the regular controls, we offered all available therapeutic options. Because of the missing tumor growth and the moderate clinical complaints that could be compensated by hearing aids regarding the hearing ability, the patient finally decided again for regular tumor observation in the sense of wait-and-scan.
- Over 5 years, no significant tumor growth was observed without therapy (Figure 38 (Fig. 38)).

#### Stage 2

**Functional hearing ability**

**Case #3, male, age: 73 years**

History:

- First presentation in January 2007, at that time recurrent rotational vertigo had occurred for 5 years.
- Increasing hearing loss on the left side for 2 years and temporal pressure sensation on the left side.
- Otherwise inconspicuous anamnesis.

Clinical findings:

- High-frequency hearing loss on the healthy side to max. 40 dB at 6 kHz.
- On the left side, steep slope of 20 dB at 1 kHz up to a maximum of 70 dB at 6 kHz.
- The facial and vestibular nerves were inconspicuous.

MRI:

- The images of an externally performed MRI showed a mainly intrameatal and slightly extrameatal vestibular schwannoma with a maximum length of 10 mm.

Intervention:

- Wait-and-scan.

**Figure 39:**
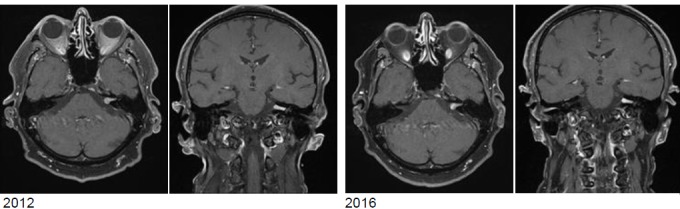
Axial and coronal MRI; T1–w + CM . Mostly constant size of a vestibular schwannoma during an observation time of 3.5 years.

Course:

- Regular control examinations first revealed only a slight growth of the extrameatal part of the schwannoma over 5 years (until 2012) up to a maximum size of 13x0.4x0.7 mm as well as slight progression of the left-sided hearing loss.
- No functional deficit of the vestibular nerve, although the balance tests revealed discrete signs of peripheral vestibular disorder on the left side.
- The patient decided for further observation of the findings.
- Annually performed examinations showed constant growth of the tumor over 3.5 years (last examination performed in March 2016).
- Audiology revealed a slight increasing hearing loss up to now 95 dB at 4 kHz with constantly good balance function (Figure 39 (Fig. 39)).

## 10 Classification for decision-making regarding treatment and treatment pathways

Up to now it was not possible to define a multicenter, multidisciplinary consensus for medical management of patients suffering from vestibular schwannomas. The decision-finding diagram is meant to be a contribution to the discussion. Similar algorithms have turned out to be a good basis for decision-making in the context of tumor board.

In the decision-making process, the size of the tumor, its growth rate, the possibility or probability of complete resection with preservation of the hearing function and the function of the facial nerve, the patient’s age and previous diseases, the possibly good management of factors like vertigo and tinnitus that are very important for the quality of life, and finally also the patient’s opinion play a crucial role. Finally, also the experience and the outcomes of the therapy center regarding the different treatment modalities influence the decision. Before discussing the treatment modality, the expert team should clarify the question if a therapy of the tumor at the actual time is reasonable or if further observation including imaging procedures at a certain time may be the best option of the patient.

Table 2 (Tab. 2) describes a possible matrix for the treatment of a patient with vestibular schwannoma. In order to base a treatment on such a matrix, unbiased consideration and sincere assessment of own treatment results are required. Here, the example of a 75-year-old patient with functional hearing is displayed who has an intra-/extrameatal tumor confirmed by MRI that reaches into th cochlear fossa. The probability that the treatment of such a patient leads to immediate or delayed morbidity, is not low. Furthermore, the growth rate of the tumor is unclear and it cannot be excluded that the vestibular schwannoma will have a constant size over several years. The tumor board would then decide in this case of first manifestation to perform MRI again after 6 months.

In the context of this example, observation of the tumor is expected to achieve the best result for the patient.

A patient with occlusive hydrocephalus (stage 4b according to Samii [9]) will certainly be recommended to undergo (partial) resection of the tumor, regardless the age, whereas for an old patient with communicating, non-resorptive hydrocephalus the tumor might be observed after application of a shunt system in the ventricular system. 

Cystic vestibular schwannomas may be one of the most demanding challenges of microsurgical tumor resection, also because the affected nerves can be highly split up and run adherently at the cell walls so that also larger vessels are difficult to be separated from tumors (see also case series [37]).

**Figure 40:**
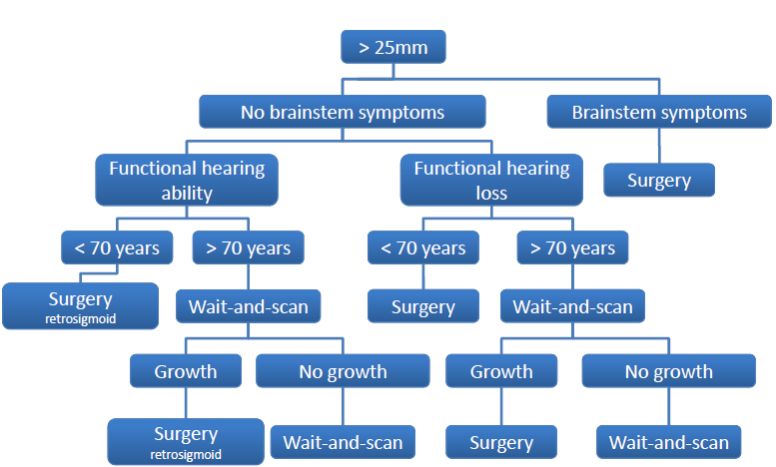
Decision algorithm for patients with a tumor diameter of more than 25 mm.

**Figure 41:**
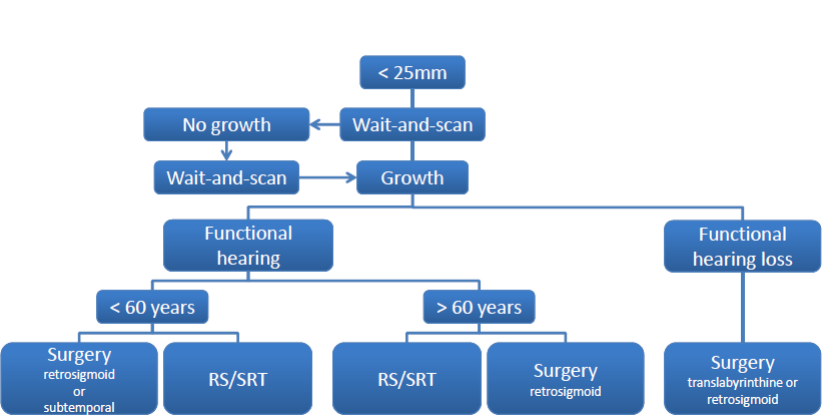
Decision algorithm for patients with a tumor diameter of less than 25 mm.

The following decision-finding diagrams (Figure 40 (Fig. 40) and Figure 41 (Fig. 41)) simplify enormously, but they include relevant criteria and may be helpful for decision-making. Also in this context, individual factors play a crucial role; none of the described decision-finding schemes is meant to be a dogma. It is more appropriate to consider for example the volume of a tumor as criterion instead of the largest diameter, especially in cases of irregular tumors [38]. The more complex measurement of the volume is not available in all centers so that we mention here the highest tumor diameter as criterion. Especially in neurosurgical series, mostly tumors of stage 3 and 4 are found. A primarily radiosurgical or stereotactic radiotherapy for tumors with a diameter of more than 25 mm is problematic because of the frequently occurring post-interventional swelling of the tumor and the possible development of a severe peri-lesional edema [39]. Our treatment pathways do not provide such a therapeutic option for tumors measuring more than 25 mm in diameter. If already at the time of first presentation symptoms exist that are due to brainstem compression, further observation is certainly no reasonable option (Figure 40 (Fig. 40)).

Tumors with a maximal diameter of less than 25 mm may be observed to draw conclusions on their biological growth behavior (Figure 41 (Fig. 41)). This may be a prognostic factor for hearing preservation as well as for the probability of recurrence development after incomplete resection.

## 11 Quality of life and preservation of the function

The quality of life of patients with vestibular schwannomas depends on a multitude of different factors. Therapeutic interventions, in particular surgeries, are often associated with a sudden break regarding the quality of life that disappears usually in the further course. Thus it is already decisive for the result of such a survey to determine the time of assessing the quality of life. The scales applied for the evaluation of the quality of life are not homogeneous and partly not even specific for this disease. Meanwhile, however, validated specific questionnaires are available for the disease such as for example the Penn Acoustic Neuroma Quality of Life questionnaire (PANQOL) [40]. In a prospective study, Carlson et al. quantify the smallest VAS difference that is perceived as significant by the patient in the PANQOL for various symptoms in the following way: PANQOL overall score: 11 points; hearing: 6 points; balance: 16 points; paresis of the facial nerve: 10 points; pains: 11 points; energy: 13 points; anxiety: 11 points; general well-being: 15 points [41].

The weighting of the single factors influencing the quality of life of patients varies enormously. Vertigo plays a key role as well as an increasing hearing loss associated with social restrictions [42]. In this context it is mostly irrelevant which primary management strategy is pursued. A recent study (538 patients; 46% stereotactic irradiation, 27% surgery, 28% wait-and-scan) considered vertigo as significant symptom for the long-term quality of life [43]. Astonishingly, persisting headaches were more relevant for the patients of this study regarding the quality of life than function of the facial nerve, hearing loss, and tinnitus. 

### 11.1 Preservation of the facial nerve

In a meta-analysis including more than 25,000 patients, Sughrue et al. found a reported rate of preserved facial nerves of 85% after middle fossa approach, 81% after translabyrinthine approach, and 78% after retrosigmoid approach [44]. Since the risk of damage depends on the tumor size, its configuration, and the patient’s age, significant differences in the evaluated patient populations have to be taken into account.

Looking at more homogeneous cross-over studies with all tumor stages, generally in more than 90% of the cases preservation of the facial nerve is anatomically and functionally possible in the context of complete tumor resection [45], [46], [47]. At this point, it is worth mentioning a retrospective article from Erlangen published in 2001. Hereby, the authors could report about surgical treatment of 735 patients with a middle fossa approach. In this group, the average rate of preservation of the facial nerve (House-Brackmann stages I and II) amounted to 92% [48]. Already in 1997, Samii et al. could anatomically preserve the facial nerve in 930 of 1,000 patients [45]. In another study with 200 patients, the same surgeon reported about a preservation rate of the facial nerve of 98.5% after 98 complete tumor resections [46]. In cases of very extended tumors with a maximum diameter of more than 4 cm, the functional preservation of the nerve (House-Brackmann grade I-II) was successful only in 75% (n=50 [47]). Persisting paresis of the facial nerve after unilateral (radiosurgery) or fractionated stereotactic irradiation are rather rare with 1–4% [49], [50], [51]. Regarding hypofractionated radiotherapy of tumors with a diameter of >3 cm, the results with persisting high-grade paresis of the facial nerve (House-Brackmann grade III and higher) of 12 and 20% despite irradiation with a median follow-up interval of 97 months can no longer be accepted [52].

#### 11.2 Hearing preservation

Already at the time of diagnosis, many patients do no longer dispose of functional hearing on the tumor side. In a recent study with partly observed (n=148, 28%), partly microsurgically treated (n=144, 27%), or with gamma knife (n=247, 46%) treated patients with a tumor smaller than 3 cm in diameter had a functional hearing ability on the affected side in about half of the cases (n=287, 55%) [53]. Eight years after diagnosis, this rate had decreased to less than 25% of all patients (n=105, 23%). The rate of hearing preservation in this study was highest for observed patients (68%), followed by patients with stereotactic irradiation (40%), and microsurgically treated patients (14%).

Especially in cases of small tumors, however, an experienced surgeon has a good chance to completely resect the tumor and at the same time to permanently preserve the hearing ability in the affected ear [46]. For tumors of stage T1, the rate of functional hearing preservation – merely estimated based on tone audiometry – amounted to 60% of all patients; for stage T2 to 72%. In cases of T3 and T4 tumors, the rate decreased to 43%. Other authors of microsurgical series report about functional hearing preservation (pure tone audiogram with an average of <50 dB, speech discrimination >50%) in 15–40% of the cases [54], [55], [56], [57]. Also a meta-analysis (1,073 patients) showed that the hearing preservation rate in cases of intracanalicular tumors (56.9%) was better than for tumors with an extrameatal diameter of 1–9 mm (45.6%) and a diameter of 10–19 mm (32.3%) [58][58]. Regarding the volumetric determination of the tumor size, the same trend is observed with a functional hearing preservation rate of 48% for patients with a tumor volume of <2 cm³ and only 25% for patients with a tumor volume between 2 and 4 cm³ [57].

Even very large tumors (>4 cm in maximum diameter) may be operated in single cases with preservation of the hearing ability [47]. Hence, it is also reasonable in those cases to select a surgical approach that generally allows hearing preservation. An extension of the tumor into the cochlear fossa is unfavorable regarding prognostic hearing preservation. When this region in the fundus of the internal acoustic meatus was affected by the tumor, the hearing preservation rate decreased to 47% in comparison to 76% for more medially located tumors [59]. Microsurgically, there are generally 2 approaches with which hearing preservation is possible: the retrosigmoid (lateral-suboccipital) and the subtemporal approach.

#### 11.3 Vertigo

Vertigo is a symptom that may strongly influence the patient’s quality of life. Only in the last years, it has been placed into the focus of attention [60]. Actually, the characteristics of this symptom are a predictor for the ability to work, again depending on the fact if microsurgical treatment or gamma knife surgery had been performed (2013). Apart from vertigo, in the cited study no other symptom – not even paresis of the facial nerve, hearing loss, or tinnitus – correlated significantly with permanent dependence of social benefits [61]. In a prospective study with 434 cases, Andersen et al. (2015) reported that 9% of the patients with unilateral vestibular schwannomas described severe vertigo with more than 75 of 100 points on the VAS [62]. Missing caloric irritability as well as gait and posture disorders were proportional to the tumor size. In the longer time course, the symptom of vertigo often regresses, probably due to the increasingly missing afferent nerve pathways from the unilateral balance organ [42]. Independent from this fact, further tumor growth may increase tinnitus and also unsteady gait (brainstem compression, hydrocephalus). Patients whose main problem at the time of first diagnosis was already most severe vertigo, reported about clearly increasing symptoms for 3 months after after removal of intracanalicular tumors via a retrosigmoid approach, but after one year the situation had significantly improved compared to the initial findings [63]. 

In 2014, in the context of a cooperative cross-sectional investigation (Mayo, Rochester, and Bergen, Norway), 538 patients with unilateral vestibular schwannoma were asked regarding the symptom of vertigo (Dizziness Handicap Inventory, DHI) [64]. The survey was performed after an average of 7.7 years after first diagnosis. More than half of the patients reported to still suffer from vertigo after that time. Factors associated with a clearly permanent and even severer vertigo were: female gender, higher age of life, tumor size, previous headaches and migraine as well as vestibular symptoms before treatment. Further, persisting vertigo occurred in correlation with the frequency and severity of headaches after treatment. The DHI scores were independent from the aspect if patients had undergone surgery, irradiation, or wait-and-scan.

#### 11.4 Tinnitus

The incidence of tinnitus is higher in patients who still have hearing abilities in the affected ear [13]. The occurrence of tinnitus is statistically associated with the tumor growth [42]. Even if the complete removal of a vestibular schwannoma is successful with preservation of the functional hearing ability, the quality of life can be negatively influenced by a tinnitus, newly appearing or deteriorating after surgery [65]. Among 242 patients with vestibular schwannoma included in a survey, 70.7% complained about tinnitus before surgery [66]. After tumor resection via a retrosigmoid approach, the symptom had completely disappeared in 25.5% of the patients, 33.3% had an improved situation, 31.6% observed an unchanged condition, and 9.9% had severer or newly occurring tinnitus.

In the same study of 171 patients with preoperative tinnitus, the cochlear nerve was resected in 85 cases (49.7%) and in 86 (50.03%) it was preserved. Between both groups, there was no significant difference with regard to the presence of postoperative tinnitus. A clear pathophysiological explanation of the phenomenon of tinnitus in patients with vestibular schwannoma has not yet been found. 

#### 11.5 Headaches

The least clarified up to now is the role of headache and its causes. In a mixed series (46% irradiation, 27% surgery, 28% wait-and-scan) of 538 patients, Carlson et al. (2015) found headaches of variable incidence and severity in about 50% 8 years after first diagnosis. In comparison to the control group, the patients with vestibular schwannoma complained twice as much about headaches. The treatment modality did not have a significant influence on these percentages, neither had the choice of the surgical approach regarding the long-term risk of severe headaches. Younger patients, patients with higher scores on the anxiety and depression scales, and pre-existing headache diagnoses were the relevant predictors of long-term headaches, the tumor size had not significant influence [67]. It is well-known that headaches of any cause have a major impact on the quality of life. As for vertigo and tinnitus, this factor should be taken into account in the context of information and consultation of patients regarding the therapy options and the long-term treatment.

Decision-finding regarding the management of patients with vestibular schwannomas should consider the benign nature of the tumor with mostly slow growth and it should be based on the long-term preservation of the quality of life. Radical tumor resection is certainly justified when a realistic long-term benefit may be expected for the individual patient that outweighs the possible negative effect on the quality of life.

On the other hand, the stress of the patient caused by the tumor itself must not be underestimated and the chances of treatment must not be wasted by an unrealistic overemphasis of the risks

Apart from single cases, it should be the disease itself and not the decision for the treatment or the treatment modality that determines crucially the patient’s quality of life [40], [42]. Already the knowledge of the diagnosis and the natural course of the tumor disease impact the quality of life in the vast majority of the patients [35], [60], [68], [69], [70]. Therapeutic and scientific efforts should aim at preserving the quality of life of the patients suffering from this disease or even at improving it.

## 12 Neurofibromatosis type 2

Competent management of patients with the complex disease of NF2, requires a multidisciplinary team including human genetics, neurosurgery, otolaryngology, ophthalmology, neurology, pediatrics, radiology, pathology, radiotherapy, audiology, psycho-oncology, physiotherapy, and other disciplines [71]. The primary objective of medical management must be the preservation of functions because they are inseparably associated with the patients’ quality of life. For a reasonable timing of interventions regarding NF2-associated tumors it is essential to know about the growth behavior of the tumors. The tumor growth varies according to the tumor type and location [72], [73] as well as according to individual molecular genetic properties of the tumors [74], [75], [76].

Intracranially, vestibular schwannomas and meningiomas show similar average growth rates between 0.30 and 2.57 cm³ per year [72], [77], [78], [79], mostly, however, clearly less than 10% on average [80], [81]. The most frequently observed growth pattern is a saltatory growth with stable phases, sometimes for years, and growth episodes [7], [72]. The treatment results must be evaluated based on those data.

The indication for treatment and definition of the extent of the surgical resection follows more complex criteria compared to spontaneous vestibular schwannomas. Relevant factors hereby are the bilateral hearing function, tumor size, tumor extent in direction of the brainstem and the internal auditory meatus, growth rate, the patient’s preferences, and comorbidities. The benefit-risk ratio is clearly defined as the elimination of the functional threat vs. the risk of functional loss by the intervention. Even if surgery of the tumors in NF2 patients is sometimes difficult and complicated, radiotherapy is often no real alternative. Already during 5-years intervals, the tumor control rates (= growth stop and/or shrinking) vary significantly after radiation, probably also independent from the applied radiation dose, between 66 and 100%. Also the hearing preservation rate in those intervals amounts to variable 33–57% [82], [83], [84], [85], [86], [87], [88], [89], [90]. A growth stop induced by radiotherapy in NF2 patients is rarely achieved in comparison to sporadic tumors [91] and especially higher – regarding tumor control more effective – radiation doses can already accelerate the hearing loss in some very few tumors [92]. In cases of NF2, the alternative option of chemotherapy with the VEGF (vascular endothelial grow factor) inhibitor Bevacizumab is useful in some phases. This monoclonal antibody that is applied in Germany as off-label procedure can stabilize the size of vestibular schwannomas over periods of several years and even reduce it [93], [94]. In some cases, the treatment is accompanied by a stabilization or improvement of the hearing ability [95], [96]. The side effects, in particular arterial hypertension and nephrotoxicity, must not be neglected in the long run [97].

**Figure 42:**
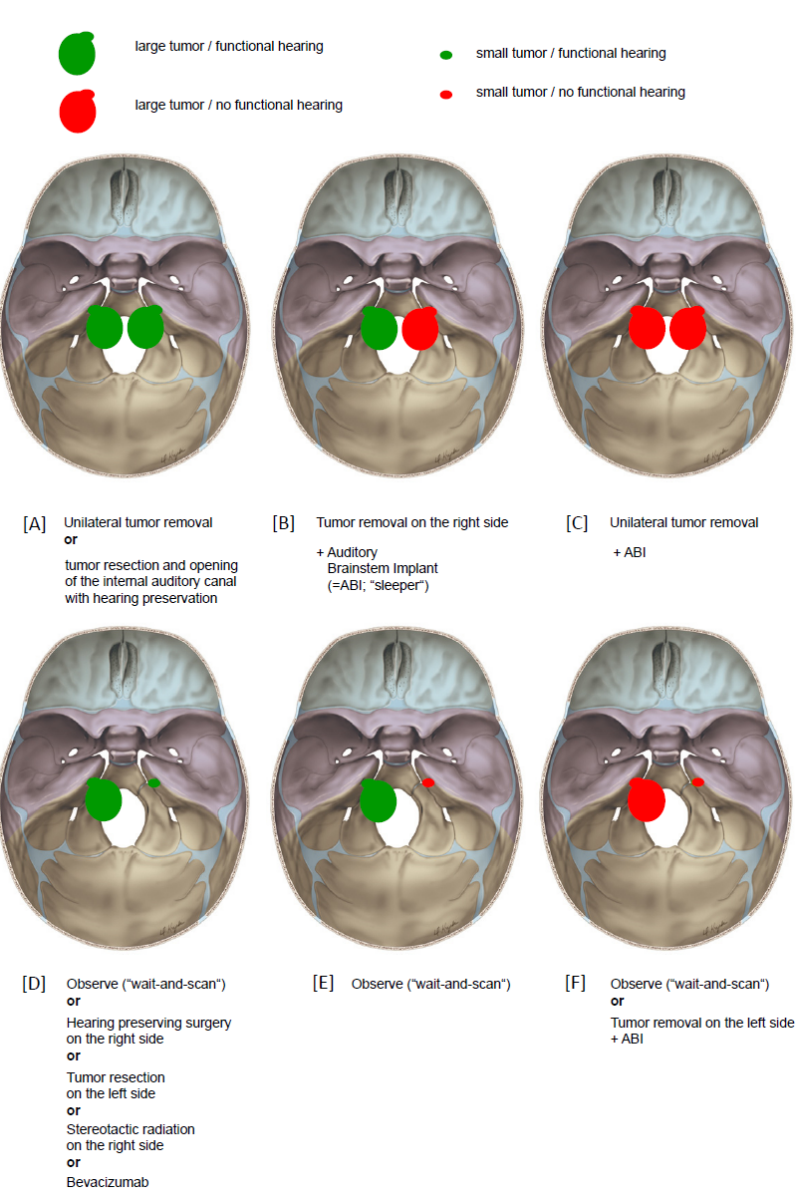
Possible constellations and treatment options in the management of vestibular schwannomas with neurofibromatosis type 2; A bilaterally large tumors and bilaterally functional hearing ability; B bilaterally large tumors and only unilaterally functional hearing ability; C bilaterally large tumors and bilaterally no functional hearing ability; D different tumor sizes and bilaterally functional hearing ability; E significantly different tumor sizes and hearing loss on the side of the smaller tumor; F significantly different tumor sizes and bilateral hearing loss.

Relevant constellations regarding the management in cases of bilateral vestibular schwannomas are displayed in Figure 42 (Fig. 42). Of course, there are single cases where others than the described management options; the individually adapted strategy requires the involvement of the complete medical and social situation of the patient.

As long as a conservative management is reasonably possible, it will generally be preferred in the sense of long-term functional preservation unless there is the microsurgical possibility to remove a small, strategically well located tumor in a bilaterally hearing patient with hearing preservation [20], [98], [99].

In cases of brainstem compressing vestibular schwannomas on both sides and bilaterally functional hearing, in general first a unilateral hearing preserving tumor reduction may be conceived (Figure 42A (Fig. 42)). If the hearing ability gets lost during surgery (intraoperative monitoring of evoked brainstem potentials), it is useful to change the strategy and to possibly achieve a complete tumor resection with preservation of the facial nerve function. This would also be the procedure when already before surgery unilateral deafness was found (Figure 42B (Fig. 42)). In those cases, it is also useful to discuss with the patient if an auditory brainstem implant should be implanted as so-called “sleeper”. If the electric conductivity of the cochlear nerve is preserved, it is generally possible – similar to patients with unilateral spontaneous vestibular schwannoma – to achieve hearing rehabilitation by means of cochlear implantation.

While in cases of unilateral or bilateral small vestibular schwannomas, generally wait-and-scan strategy is recommended, it might be useful and also possible in exceptional cases to microsurgically explore such a small tumor and to remove it with hearing preservation (Figure 42D (Fig. 42)). Of course further valid treatment scenarios are imaginable, depending on the individual situation of the patient.

In cases of large tumors and still functional hearing in one ear and a small tumor with deafness on the contralateral side, it is reasonable to wait as long as the hearing ability remains stable. If the inner ear hearing loss increases in such a case of a growing large tumor, a tumor reduction with hearing preservation, the removal of the posterior wall of the internal auditory meatus, or a therapeutic attempt with Bevacizumab are valid options.

If surgery with functional preservation of the facial nerve is not successful in a patient with neurofibromatosis type 2, an immediate transplantation in the same session allows for the best chances for regeneration. A side-to-end connection between the hypoglossal and the facial nerves within the first year after paralysis of the facial nerve can still lead to an equally good result regarding rehabilitation of the face [100], [101], [102], [103], [104]. For longer lasting pareses, plastic reconstructions with dynamic temporal muscle transfer, tarsorrhaphy, and eyelid implantations (lid loading) must be discussed. The hearing rehabilitation can be performed in patients with still acoustically useable hearing first with conventional hearing aids. If they are no longer sufficient, the implantation of a cochlear implant (CI; possible in cases of intact hearing nerve and bipolar neurons in the cochlea) or of an ABI (auditory brainstem implant) can be considered according to the individual dynamics of the vestibular schwannoma.

## Notes

### Competing interests

The authors declare that they have no competing interests.

## Figures and Tables

**Table 1:**
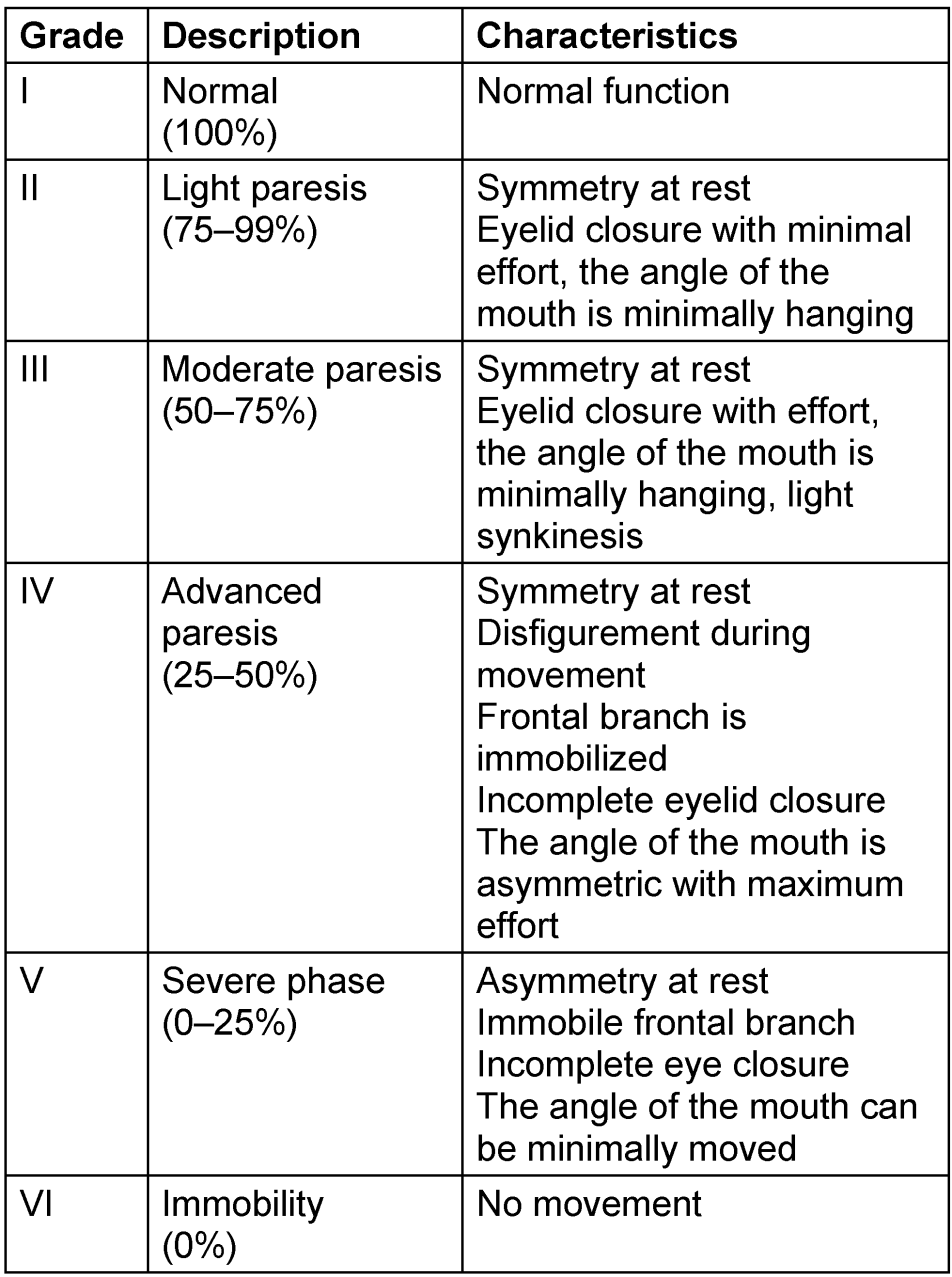
Grading of the facial nerve function, simplified according to House & Brackmann [105] mentioning the percentage of the deficit.

**Table 2:**
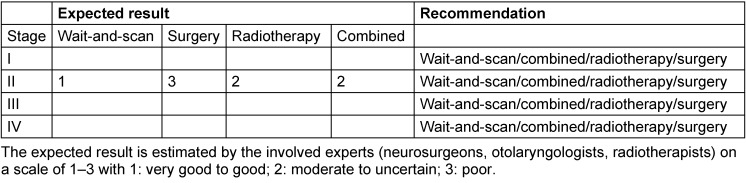
Decision matrix with hypothetical data on the treatment of a patient
